# Genome-wide analysis of three-way interplay among gene expression, cancer cell invasion and anti-cancer compound sensitivity

**DOI:** 10.1186/1741-7015-11-106

**Published:** 2013-04-16

**Authors:** Yi-Chiung Hsu, Hsuan-Yu Chen, Shinsheng Yuan, Sung-Liang Yu, Chia-Hung Lin, Guani Wu, Pan-Chyr Yang, Ker-Chau Li

**Affiliations:** 1 Institute of Statistical Science, Academia Sinica, 128 Academia Road, Section 2, Taipei, 115, Taiwan; 2 NTU Research Center for Medical Excellence - Division of Genomic Medicine, 2 Syu-Jhou Road, Taipei, 100, Taiwan; 3 Department of Clinical Laboratory Sciences and Medical Biotechnology, National Taiwan University College of Medicine, 1 Chang-Te Street, Taipei, 100, Taiwan; 4 Department of Internal Medicine, National Taiwan University College of Medicine, 1 Jen-Ai Road, Taipei, 100, Taiwan; 5 Institute of Biomedical Sciences Academia Sinica, 128 Academia Road, Section 2, Taipei, 115, Taiwan; 6 Department of Statistics, University of California, Los Angeles, Los Angeles, CA, 90095, USA

**Keywords:** NCI-60, Invasion, Metastasis, Microarray, Chemotherapy

## Abstract

**Background:**

Chemosensitivity and tumor metastasis are two primary issues in cancer management. Cancer cells often exhibit a wide range of sensitivity to anti-cancer compounds. To gain insight on the genetic mechanism of drug sensitivity, one powerful approach is to employ the panel of 60 human cancer cell lines developed by the National Cancer Institute (NCI). Cancer cells also show a broad range of invasion ability. However, a genome-wide portrait on the contributing molecular factors to invasion heterogeneity is lacking.

**Methods:**

Our lab performed an invasion assay on the NCI-60 panel. We identified invasion-associated (IA) genes by correlating our invasion profiling data with the Affymetrix gene expression data on NCI-60. We then employed the recently released chemosensitivity data of 99 anti-cancer drugs of known mechanism to investigate the gene-drug correlation, focusing on the IA genes. Afterwards, we collected data from four independent drug-testing experiments to validate our findings on compound response prediction. Finally, we obtained published clinical and molecular data from two recent adjuvant chemotherapy cohorts, one on lung cancer and one on breast cancer, to test the performance of our gene signature for patient outcome prediction.

**Results:**

First, we found 633 IA genes from the invasion-gene expression correlation study. Then, for each of the 99 drugs, we obtained a subset of IA genes whose expression levels correlated with drug-sensitivity profiles. We identified a set of eight genes (*EGFR, ITGA3, MYLK, RAI14, AHNAK, GLS, IL32* and *NNMT*) showing significant gene-drug correlation with paclitaxel, docetaxel, erlotinib, everolimus and dasatinib. This eight-gene signature (derived from NCI-60) for chemosensitivity prediction was validated by a total of 107 independent drug tests on 78 tumor cell lines, most of which were outside of the NCI-60 panel. The eight-gene signature predicted relapse-free survival for the lung and breast cancer patients (log-rank *P* = 0.0263; 0.00021). Multivariate Cox regression yielded a hazard ratio of our signature of 5.33 (95% CI = 1.76 to 16.1) and 1.81 (95% CI = 1.19 to 2.76) respectively. The eight-gene signature features the cancer hallmark epidermal growth factor receptor (EGFR) and genes involved in cell adhesion, migration, invasion, tumor growth and progression.

**Conclusions:**

Our study sheds light on the intricate three-way interplay among gene expression, invasion and compound-sensitivity. We report the finding of a unique signature that predicts chemotherapy survival for both lung and breast cancer. Augmenting the NCI-60 model with *in vitro* characterization of important phenotype-like invasion potential is a cost-effective approach to power the genomic chemosensitivity analysis.

## Background

Gene-expression profiling of tumors from patient cohorts has been used to develop gene signatures for clinical outcome prediction. Recently, a signature combining estrogen receptor (ER) status and predicted chemo/endocrine responsiveness succeeded in identifying patients with high probability of survival following taxane and anthracycline chemotherapy [1]. However, the biological mechanism of the genes involved in such cohort-initiated genomic predictors may not be easy to elucidate. On the other hand, *in vitro* chemosensitivity experiments on cancer cell lines, such as the NCI-60 cell line panel, are helpful in elucidating the complex relationship between drug responsiveness and gene expression. Despite this, the more challenging problem of how to translate the elucidated relationship for clinical outcome prediction still awaits more studies.

In addition to chemosensitivity, metastasis is another major issue in studying treatment efficacy for many cancers, including invasive breast and lung cancer. Like the drug-sensitivity heterogeneity, tumor cells often exhibit a wide range of invasion ability. Such invasion heterogeneity may exist not only between different cancer types, but also among the individual cells from the same malignant neoplasm of a patient. More subtly, it is possible that the clinical outcome of chemotherapy may hinge on the growth-inhibition of the more invasive cells rather than the less invasive cells in the neoplasm. However, a characterization of genes associated with both the invasion potential and drug-sensitivity heterogeneity is lacking.

There are four aims in this study:

(a) Molecular markers of tumor invasion potential: to identify the set of IA genes whose expression levels are likely to be indicative of the invasion potential of a tumor;

(b) Drug sensitivity prediction by tumor-invasion markers: to evaluate how the expression levels of IA genes in a tumor are likely to be indicative of the tumor’s resistance or responsiveness to an anti-cancer drug;

(c) Drug discovery with predictable sensitivity: to find anti-cancer drugs whose efficacies correlate with tumor-invasion potential and can be predicted by tumor-invasion markers;

(d) Clinical validation: to demonstrate the use of the IA gene signature for predicting clinical outcome.

NCI-60 is a diverse panel of 60 cell lines used by the Development Therapeutics Program (DTP) of the National Cancer Institute (NCI) to screen more than 100,000 compounds since 1990 [2-5]. These human cancer cell lines are derived from patients with leukemia, melanoma, lung cancer, colon cancer, central nervous system cancer, ovarian cancer, renal cancer, breast cancer and prostate cancers. The molecular characteristics of these cell lines have been subjected to various DNA microarray studies using both Affymetrix (Santa Clara, CA, USA) and spotted cDNA array technology [2].

Both drug sensitivity and gene expression profiles of the NCI-60 panel are available from the public domain; however, there is no public invasion phenotype data for NCI-60. So we conducted the invasion assay for 53 solid tumor cell lines from the NCI-60 panel in our lab.

**Figure 1:**
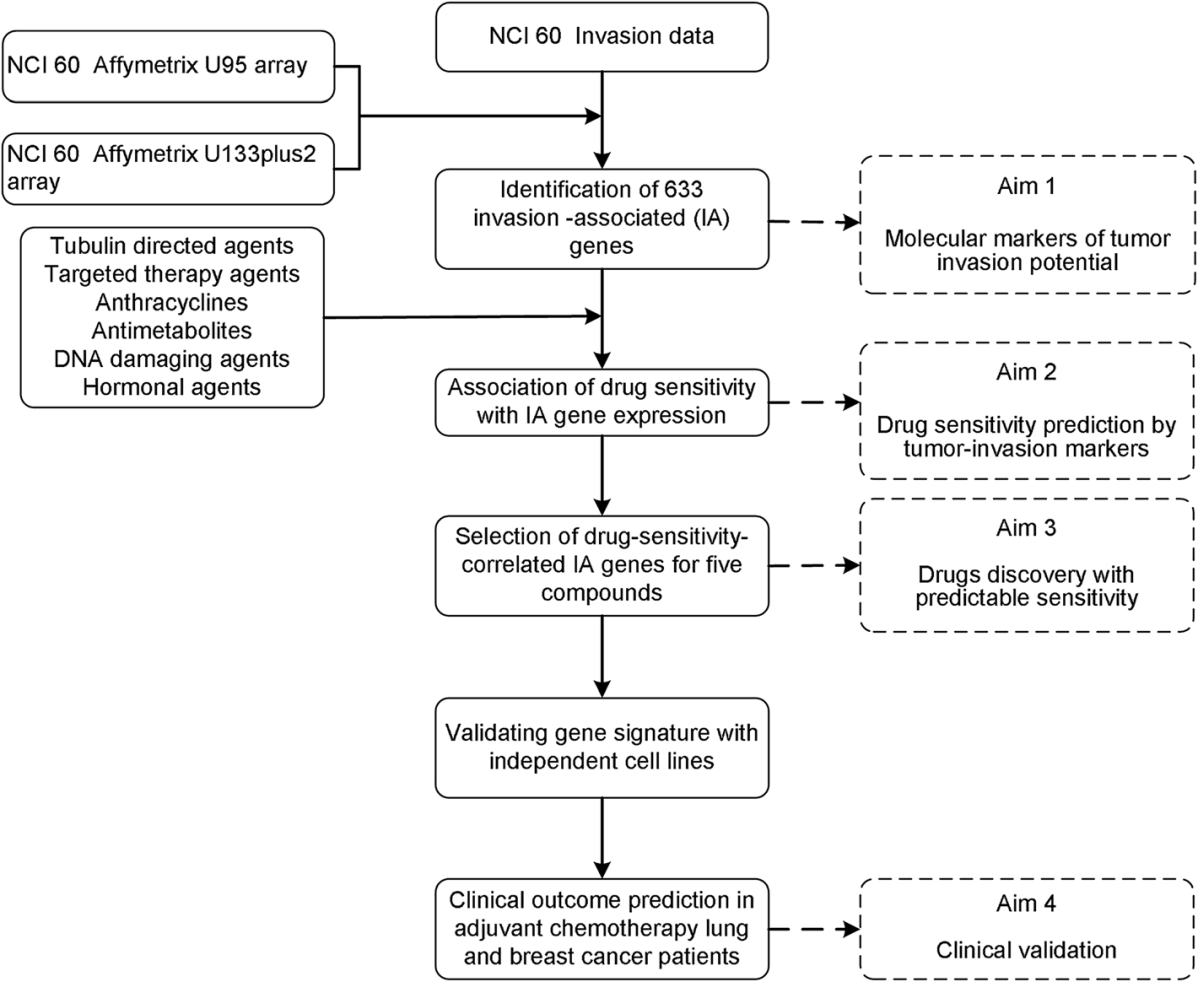
A schematic diagram illustrating the design of this study.

We then conducted a series of statistical analysis to combine information from invasion profiling, gene expression and compound-sensitivity profiling. Figure 1 outlines how we approached each of our four aims. We identified a set of 633 IA genes as the likely marker candidates of tumor invasion potential. For each of the 99 anticancer compounds of known mechanism, we studied the gene-drug correlation to identify IA genes that can be predictive of a cell’s responsiveness to the compound. A final set of eight IA genes for chemosensitivity prediction on five selected compounds was obtained. We then validated the gene signature with additional cell lines. To show the clinical relevance of our finding, we searched for published chemotherapy clinical cohorts with related regimens to test the performance of our gene signature. We found two recent studies containing anti-microtubule chemotherapy, one on lung cancer and the other on breast cancer. Our signature succeeded in predicting the clinical outcome for both cohorts.

## Methods

### Matrigel invasion assay

We purchased the panel of NCI human cancer cell lines (NCI-60) to conduct tumor invasion assays. The suspension leukemia cancer cell lines were excluded. All cells were grown in tissue culture flasks at 37°C in 5% CO_2_ in RPMI 1640 with 2 mM L-glutamine, and 10% fetal bovine serum, all from Invitrogen, Eugene, OR. The invasion capacities for cell lines were examined by using membrane invasion culture system. The HTS FluoroBlok inserts containing 8-μm pores (Falcon, Becton Dickinson, Franklin Lakes, NJ) were coated 30 μg matrigel (BD Biosciences, San Jose, CA, USA). The cells were suspended in RPMI containing 10% FBS and seeded into the upper wells of the chamber (2.5 × 10^4^ cells/well). After incubating for 24 hours at 37°C, the membrane of the transwell was fixed for 10 minutes at room temperature with methanol and stained for 30 minutes with 50 μg/ml propidium iodide (Sigma, St. Louis, MO). The number of cells in each blot was counted under a microscope with Analytical Imaging Station system (Imaging Research Inc., St. Catharines, ON, Canada). For each cell line, we reported the average invasion cell count after three repeats (n = 3).

### Cell line gene expression data

Two gene expression datasets for NCI-60 cell lines, produced by Gene Logic (Gaithersburg, MD, USA) using Affymetrix U95, and U133plus2, respectively, were downloaded from the DTP [6]. Probe mapping between U133plus2 and U95 was provided by Affymetrix.

The independent gene expression data of 78 cell lines used in the drug response validation were obtained either from the GEO website, GSE6569 (n = 23, breast cancer) [7], GSE9633 (n = 16, prostate cancer) [8], and GSE4127 (n = 29, lung cancer) [9] or by e-mail request (n = 10, lung, Balko *et al*.) [10]. All but nine cell lines were outside of the NCI-60 panel (Additional file 1: Table S1).

### Chemosensitivity data

We downloaded the NCI-60 chemosensitivity data for anti-cancer drugs with known molecular mechanisms, including those used in targeted therapy [5]. Drug sensitivity was measured by the negative of log10 GI50 (−logGI50). Seven compounds, inactive in all cell lines, were excluded. A total of 99 drugs were analyzed.

The drug sensitivity in the 78 independent cell lines was measured either by -logGI50 value [9] or by the negative of log10 half maximum inhibitory concentration (IC50) value [7,8,10].

### The real-time reverse transcription quantitative PCR analysis

The expressions of eight signature genes and a control gene *TBP* in nine lung cancer cell lines of NCI-60 were measured by reverse transcription qPCR(RT-qPCR) with specific Taqman probes and primer sets (Additional file 1: Table S2). The transcripts were amplified with Taqman One-Step RT-PCR Master Mix Reagent (Applied Biosystems) and a detection system (ABI Prism 7900HT, Applied Biosystems). Gene expression was quantified in relation to the expression of the control gene with the use of sequence detector software and the relative quantification method (Applied Biosystems).

### Statistical analysis

The comparison of the tumor invasion ability between tissue types was performed by ANOVA. We applied the normal score transformation to preprocess the gene expression data before computing the Pearson correlations. The invasion-associated genes were obtained by computing the Pearson's correlation coefficients between gene expression profiles and invasion ability profile in NCI-60 cell lines. Clustering was used to order the IA probes. Student’s *t*-test was used in validating the drug sensitivity prediction of gene signatures. All statistical analyses were performed in the R language environment [11].

### Cancer chemotherapy cohorts

Two chemotherapy cohorts were used in this study. The lung cancer cohort came from JBR.10, a randomized controlled trial of adjuvant vinorelbine/cisplatin versus observation alone [12]. We used the adjuvant cisplatin/vinorelbine therapy arm where after surgery (n = 71). The breast cancer cohort of 508 patients came from a prospective multicenter study, conducted at the M. D. Anderson Cancer Center, of which the patients were those with newly diagnosed Human Epidermal Growth Factor Receptor 2 (*HER2*)–negative breast cancer under chemotherapy containing sequential taxane and anthracycline–based regimens (followed by endocrine therapy if ER positive) [1].

### Control cohorts

Four cohorts where the patients were systemically untreated after surgery were used. Three of them were the breast cancer cohorts (GSE2034 n = 286 [13], GSE7390 n = 198 [14] and GSE11121 n = 200 [15]). The fourth, a lung cancer cohort, was the OBS arm where patients were under observation after surgery (n = 62).

### Survival analysis

We calculated the patients’ risk scores from the eight IA genes and classified them into the high-risk or the low-risk groups with the mean of risk score as the threshold value. We calculated the eight-gene risk score for each patient via simple averaging:

$$\begin{array}{c} \text{Risk score}=(G_{\text{EGFR}}+G_{\text{ITGA3}}+G_{\text{NNMT}}+G_{\text{MYLK}}\\ \;+G_{\text{IL32}}+G_{\text{GLS}}+G_{\text{AHNAK}}+G_{\text{RAI14}})/8, \end{array}$$

Kaplan-Meier survival curves were obtained and compared by log-rank tests. Multivariate Cox proportional hazard regression analysis was used to evaluate independent prognostic factors, such as age, gender, tumor stage nodal status and histological grade.

## Results

### Invasion heterogeneity

**Figure 2:**
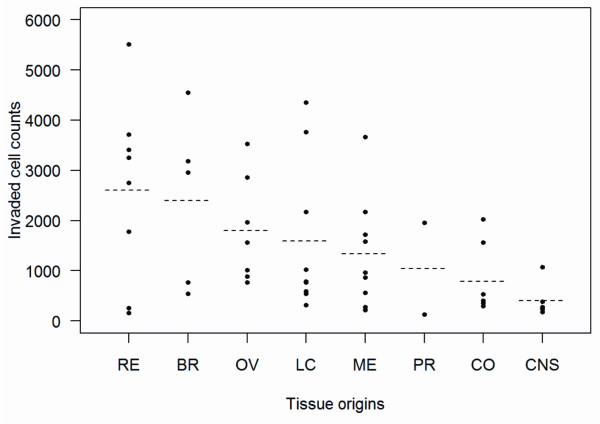
**Invasion profiling of NCI60 cancer cell lines.** Cell lines are divided into groups by their tissue origin. Each dot in each tissue group gives the invaded cell counts by the matrigel invasion assay for one cell line (n = 3). Dotted lines indicate the mean of invasion cell counts for all cell lines in each tissue group.

The matrigel invasion assay of the 53 NCI-60 solid tumor cell lines shows a great variation between different cell lines, with the invaded cell counts (ICC) ranging from 129 to 5,514 (Figure 2). According to the tissue origins, the 53 cancer cell lines are classified into eight groups: melanoma (ME), lung cancer (LC), colon cancer (CO), central nervous system cancer (CNS), ovarian cancer (OV), renal cancer (RE), breast cancer (BR) and prostate cancer (PR) [16-18]. Even within the same tissue group, there are substantial differences between the ICCs of different cell lines. Additional file 1: Figure S1 gives the overall distribution for the deviation of an individual cell line’s ICC from its group mean. The wide range of this distribution shows that the within-group variation means are greater than the between-group variation means. This suggests that the tissue of origin may not be an essential factor in characterizing invasion heterogeneity between cancer cell lines. We tested the equality of the ICC group means by ANOVA and found that the significance barely passes the 5% mark (*P* = 0.048).

### Identification of 633 IA genes

We identified the IA genes from Affymetrix U133plus2 NCI-60, and U95 NCI-60 microarray gene expression datasets by a two-stage procedure which required that (a) the gene expression profile must be significantly correlated with the invasion profile in both datasets, and (b) the sign of correlation must be consistent in both datasets (see the Additional file 1, Supplementary information). A total of 744 probes, which represented 633 distinct genes, were obtained. We estimated the false discovery rate (FDR) at the confirmation stage to be 0.08 (FDR = (2,417*0.025)/744; where 2,417 = total number of probes considered at the confirmation stage and 0.025 = the *P*-value cutoff with the sign consistency criterion).

**Figure 3:**
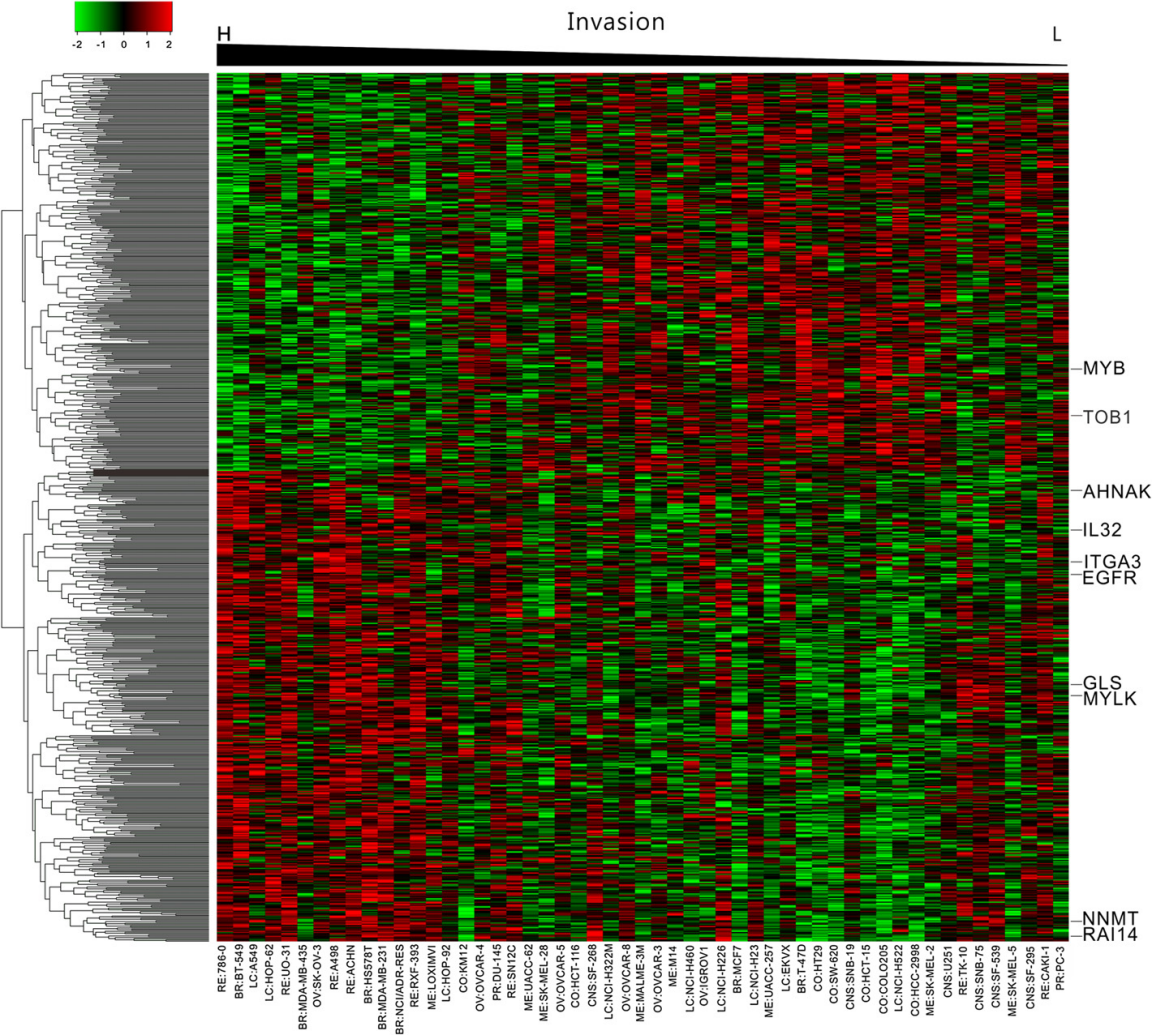
**Heat map for the expression of IA genes.** Cell lines are ordered according to the invasion ability (measured by ICC) with the highest ICC placed leftmost. The genes in the top panel have negative correlations with invasion while the genes in the bottom panel have positive correlations.

We show the expression levels of the 744 probes (Affy U133plus2) in the 53 cell lines with a heat map (Figure 3). The ordering of cell lines in this figure is based on their invasion abilities, the higher ones being on the left. The ordering of the genes is the output of the hierarchical clustering algorithm applied to the 744 expression profiles. The top panel of genes contains 341 probes that correlate negatively with the invasive ability, while the bottom panel of 403 probes correlates positively with the invasion ability.

### Functional enrichment analysis of IA genes

We use the MetaCore™ (Thomson Reuters, New York, NY), a web-based computational platform designed for system biology and drug discovery, to conduct functional enrichment analysis of IA genes. We input the set of 633 IA genes (744 probes) and used the Functional Ontology Enrichment tool with the default settings. The results show that our IA genes were enriched in cell adhesion and cytoskeleton remodeling pathways (Additional file 1: Table S3).

Additional file 1: Table S3 list the significantly enriched pathways and networks (*P* <0.0001). The results indicate the involvement of cell adhesion and cytoskeleton remolding actin cytoskeleton network (*ACTN1*, *ACTN4*, *ATCB*, *ACTG1*, *ZYX*, *VCL* and *CFL*), integrin signaling (*ITGA3*, *ITGB1*, *CAV1* and *CAV2*), matrix metalloproteinase signaling (*CDH2*, *CD44*, *TGFB2*, *TGFBR2*, *JAK1*, *SMAD1* and *SMAD3*), microtubule cytoskeleton (*TUBB* and *TUBB6*), and myosin signaling (*MYLK1*, *LIMK1*, *MYL6* and *MYH9*). We further examined the Integrin-mediated cell adhesion and migration pathway, (*P* = 5.49E-8, input/total nodes = 11/48), which has a much higher ratio of root to total nodes and significant *P*-value. The Integrin-mediated cell adhesion and migration pathway shows that ITGA3 promotes focal adhesion kinase (*FAK*) autophosphorylation and creates a binding site for c-Src. EGFR signaling also activates the FAK/Src pathway [19,20]. *FAK* activation regulates the *ERK*-mediated phosphorylation and activation of Myosin light chain kinase (*MYLK*) contributes to cell-matrix adhesion dynamics [21]. Integrin recruits *FAK* and a cytoskeletal protein vinculin and alpha-actin to focal contacts. *c-Src* and *ERK2*-mediated phosphorylation of *FAK1* promotes its release from focal contacts and *ERK2*-mediated phosphorylation of paxillin promotes the association of non-phosphorylated *FAK1* with paxillin at new or growing focal contact sites [22]. Activation of *MYLK* together with inactivation of *PAK1* contributes to cell-matrix adhesion dynamics. *PAK1* phosphorylation leads to the activation of LIM-kinase 1 (*LIMK1*) [23], inhibition of *MYLK*, activation of myosin regulatory light chains (*MRLC*) [24]. *FAK* is a tyrosine kinase which interacts with the important oncogene *c-Src*. *FAK* signaling is important for integrin regulated cell adhesion and migration. Notably, this pathway is the target of dasatinib [25], a drug we address further in this paper. A simplified version of the Integrin-mediated cell adhesion and migration pathway is shown Additional file 1: Figure S2, which features three IA genes, *EGFR, ITGA3, MYLK*.

### Association of drug sensitivity with IA gene expression

To evaluate how the abundance of the IA gene transcripts in a cancer cell correlates with the cell’s response to each of the 99 anti-cancer drugs for which the drug-sensitivity profiles on NCI-60 were available, we computed Pearson's correlation between the gene expression profile of each IA gene and the chemosensitivity profile of each compound. A positive correlation means that cell lines with higher gene expression are more sensitive to the drug, while a negative correlation indicates the opposite. The gene-drug correlation results are shown in a heat map (Additional file 1: Figure S3A). We identified all the significant correlations (*P* <0.05), and displayed the findings in Additional file 1: Figure S3B. An IA probe showing a significant correlation with a drug would be called a drug-sensitivity-correlated probe for that drug. We counted the number of drug-sensitivity-correlated IA probes for each compound.

A higher count indicates the availability of more IA genes for the sensitivity prediction of that drug. Such a drug would be more likely to have differential anti-cancer effects among the tumors showing differential invasion potential. Drugs with higher counts are this study’s primary interest.

Individually speaking, the compound with the highest count is zoledronic acid, a bisphosphonate drug used to prevent skeletal fractures in cancer patients and to treat osteoporosis. Interestingly, recent studies indicate zoledronic acid can prevent skeletal metastases through inhibition of invasion and angiogenesis of cancer cells [26,27]. However, we were unable to find the drug sensitivity validation and gene expression data outside the NCI panel for zoledronic acid.

**Figure 4:**
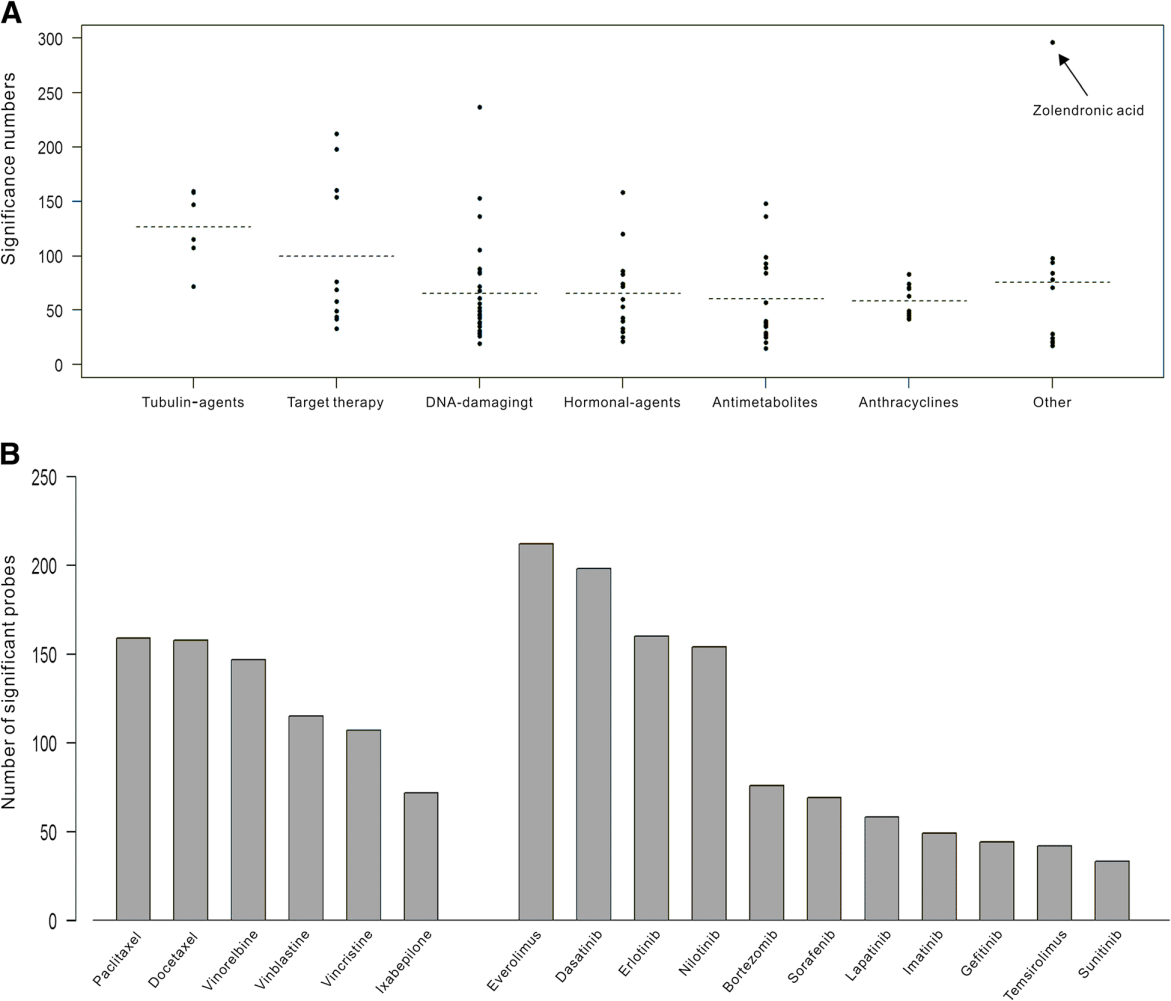
**The distribution of drug-sensitivity-correlated IA probes.** (**A**) The number of IA probes with significant (*P* <0.05) gene-drug correlation with each anti-cancer compound is plotted according to the grouping of the drug mechanism. The dotted line gives the mean of the probe counts in each group. (**B**) The numbers of significant IA probes in tubulin-binding agents and targeted therapy agents.

To investigate Additional file 1: Figure S3B further, we present each drug’s count of the drug-sensitivity-correlated IA probes by grouping according to the drug’s action mechanism (Figure 4A).

We find that on average, the group of tubulin-binding agents ranks the highest, followed by the targeted therapy. The count for each compound in these two groups is given in Figure 4B. We continued our study on the 17 drugs from these two groups.

### Gene-drug heat map for 17 drugs

**Figure 5:**
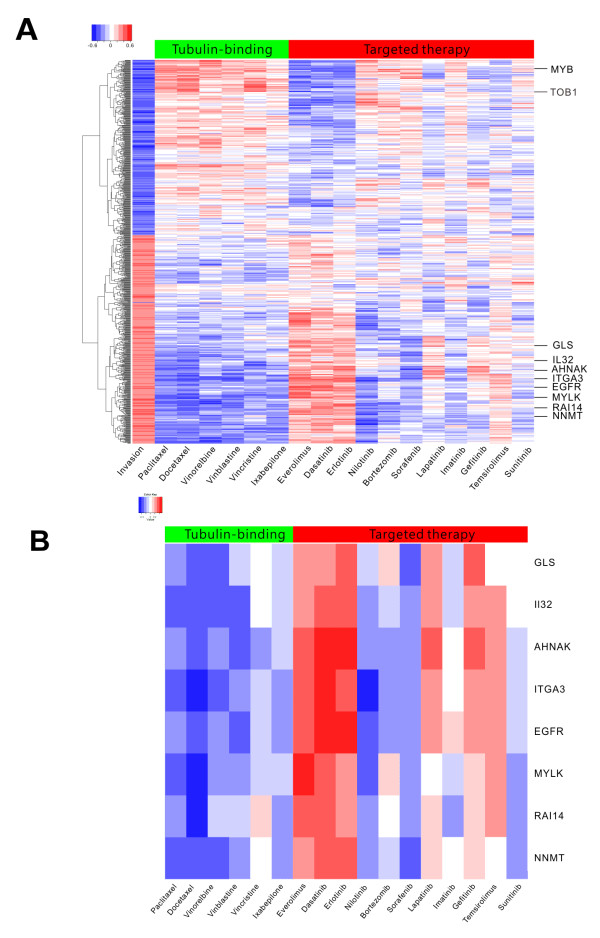
**Heatmap of gene-drug correlation.** (**A**) Heatmap showing the gene-drug correlations for tubulin-binding and targeted therapy agents. (**B**) The specific pattern for the eight-gene signature enlarged from **A**. Blue, negative correlation; red, positive correlation.

We represent the 744 by 17 gene-drug correlations as a heat map after clustering the genes (Figure 5A). We kept the ordering of compounds to be the same as in Figure 5B. Interestingly enough, we found that the top three compounds in the group of target therapy agents, everolimus, dasatinib and erlotinib, showed a color pattern almost completely opposite to the tubulin-binding agents (Figure 5). This means that cells with higher expressions of genes, such as *MYB* and *TOB1*, tend to be more sensitive to tubulin-binding agents but they also tend to be more resistant to the three-target therapy drugs. Likewise, cells with lower expression of genes, such as *EGFR* and *ITGA3*, tend to be more resistant to tubulin-binding agents but they also tend to be more sensitive to the three-target therapy drugs. In other words, the efficacy of the two groups of compounds tends to be in the opposite direction. The clustering pattern in Figure 5A can also be easily detected from the column-to-column correlations (Additional file 1: Table S4). The correlations among everolimus, dasatinib and erlotinib are much higher than other correlations in the target agents. Similarly, the correlation between paclitaxel and docetaxel is much higher than other correlations in the tubulin-binding agents.

Dasatinib is a clinically studied SRC inhibitor for cancer therapy [28]. Our functional enrichment analysis of IA genes shows that the Src signaling pathway played an important role in integrin-mediated cell adhesion and migration pathway (Additional file 1: Figure S2). Erlotinib inhibits the intracellular phosphorylation of tyrosine kinase associated with the EGFR [29]. Everolimus is an mTOR inhibitor [30]. In the anti-tubulin group, docetaxel is a semisynthetic side chain analog of paclitaxel.

### Selection of drug-sensitivity-correlated IA genes for five compounds

In addition to the mechanism sharing and the clustering pattern observed, we also noticed that the numbers of sensitivity-correlated IA genes that these five compounds had are higher than other drugs in the respective mechanism groups (Figure 4A).

This prompted us to ask if these five compounds might share any drug-sensitivity-correlated IA genes that could be used for drug-sensitivity prediction. By inspecting the drug-sensitivity-correlated IA gene list for these five compounds, 26 common probes are identified (Additional file 1: Table S5). Among them, 19 probes are in the Affy HG U-133A chip and 7 probes are in the HG U-133B chip. Because most microarray data are available in the public domain used only the U-133A chip, we exclude the seven U133-B probes. We use the standard deviation (SD) of the expression across all 53 cell lines to further exclude probes showing low expression variation. Only those probes with SD ranking within the top 10% are retained. Finally, we check the sign of correlation between gene expression and drug sensitivity to ensure the consistency in predicting sensitivity or resistance.

A final set of eight IA genes (*EGFR, AHNAK, GLS, IL32, ITGA3, MYLK, NNMT*, and *RAI14*) are obtained. We confirm the quality of the microarray gene expression data by performing the qPCR assay for these eight genes on nine lung cancer cell lines in NCI-60 (Additional file 1: Figure S4). A total of 72 data points are plotted, each dot representing the expression level of a gene measured by qPCR (horizontal axis) and the microarray (vertical axis). A significant positive trend (Pearson correlation = 0.69, 2.129e-11) is observed, confirming the consistency of the two assays.

### Biological functions of eight invasion-associated genes

The eight IA genes we identified are associated with cellular cytoskeleton, cell invasion and oncogenetic signaling, including the well-known cancer driver EGFR, and FAK-Src signaling (*ITGA3, MYLK*); *ITGA3* (integrin, alpha 3) mediates cell survival and invasion through FAK-Src signaling [31-33]. *MYLK* is a key target of FAK–Src signaling [34]. Glutaminase (GLS) was shown to be up-regulated in MYC induction cancer cells and inhibition of GLS decreased the cancer cell growth [35,36]. Retinoic acid induced 14 (RAI14) is an actin cytoskeleton protein regulated by retinoic acid [37]. High expression of interleukin 32 (IL32) was shown to cause a worse clinical outcome in lung cancer patients [38]. AHNAK nucleoprotein was reported as pseudopod-specific proteins in different metastatic human tumor cell lines [39]. The activity of nicotinamide N-methyltransferase (NNMT) in catalyzing the N-methylation of nicotinamide is important for biotransformation of drug and xenobiotic compounds [40]. NNMT was identified as a tumor biomarker [41-43], promoting cell migration [44] and cell invasion [45].

### Validating gene signature with independent cell lines

**Figure 6:**
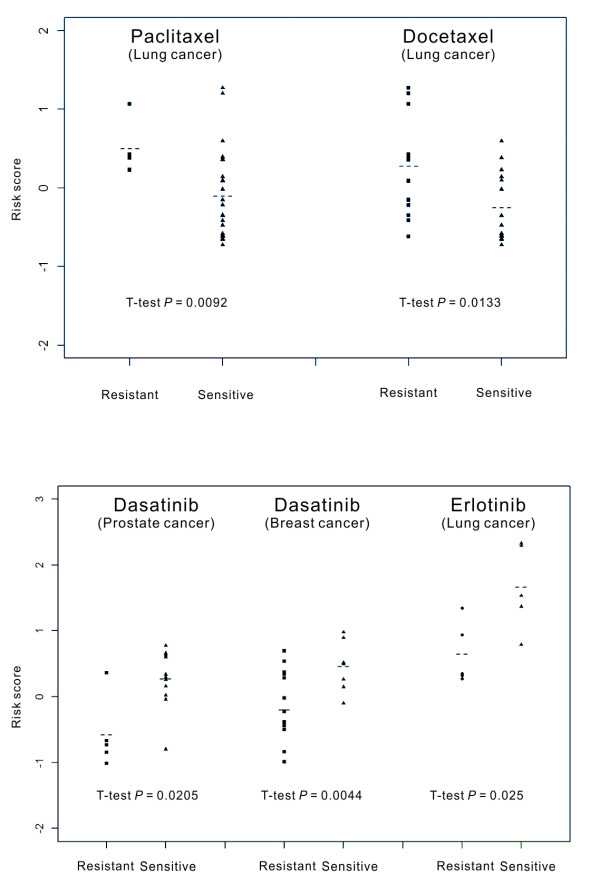
**Plots of the eight-gene risk scores between drug-sensitive and drug-resistant groups of cell lines.** The dotted line indicated the mean of each group.

We used the simple averaging to combine the expressions of eight IA genes into an eight-gene score. We searched the public domain extensively for drug response experiments involving any of the five featured compounds. Four experiments were found. In the first two experiments [9], two standard anti-microtubule agents, paclitaxel and docetaxel, were applied separately to 29 lung cell lines, which were then divided into drug-resistant and drug-sensitive groups according to the IC50 value of the experiment outcome. From the gene expression data, we calculated the eight-gene score for each cell line and found a significant difference between the drug-resistant group and the drug-sensitive group (t-test *P* = 0.009 in paclitaxel-treated group, and *P* = 0.013 in docetaxel-treated group, Figure 6). In the third experiment [8], dasatinib was applied to 16 prostate and 23 breast cancer cell lines [7], which were then divided into dasatinib-sensitive and dasatinib-resistant groups according to the experiment outcome. The eight-gene scores show a significant difference between the dasatinib-sensitive group and dasatinib-resistant group (t-test *P* = 0.0044 for breast cancer and *P* = 0.0044 for prostate cancer, Figure 6). The fourth experiment applied erlotinib to 10 lung cancer cell lines. Again, the difference between erlotinib-sensitive and erlotinib-resistant was significant (t- test *P* = 0.025, Figure 6). It should be noted, however, that these plots also showed an overlapping pattern in the distributions between the drug-resistant group and drug-sensitive group. This indicates that the eight gene biomarkers still cannot accurately classify cell lines into a resistant group versus a sensitive group.

In these validation experiments, we found nine cell lines from the NCI-60 panels. We removed these cell lines from the validation data and conducted the comparison again (Additional file 1: Figure S5). All significant differences in the first three experiments were still valid. For the fourth experiment, the *P*-value increased to 0.0788, mainly because the sample size (n = 8) was too small.

### Clinical outcome prediction in adjuvant chemotherapy lung and breast cancer patients

To test if the eight genes have the predictive power in clinical outcome evaluation, we searched the public domain for chemotherapy cohort studies involving any of the two anti-microtubule compounds we used in obtaining the eight genes. Two recently published studies on the vinorelbine containing regimen for lung cancer [12] and the taxane-containing regimen for treating invasive breast cancer [1] were obtained. We use the simple average expression of the eight-genes as the risk score to dichotomize patients into two groups.

**Figure 7:**
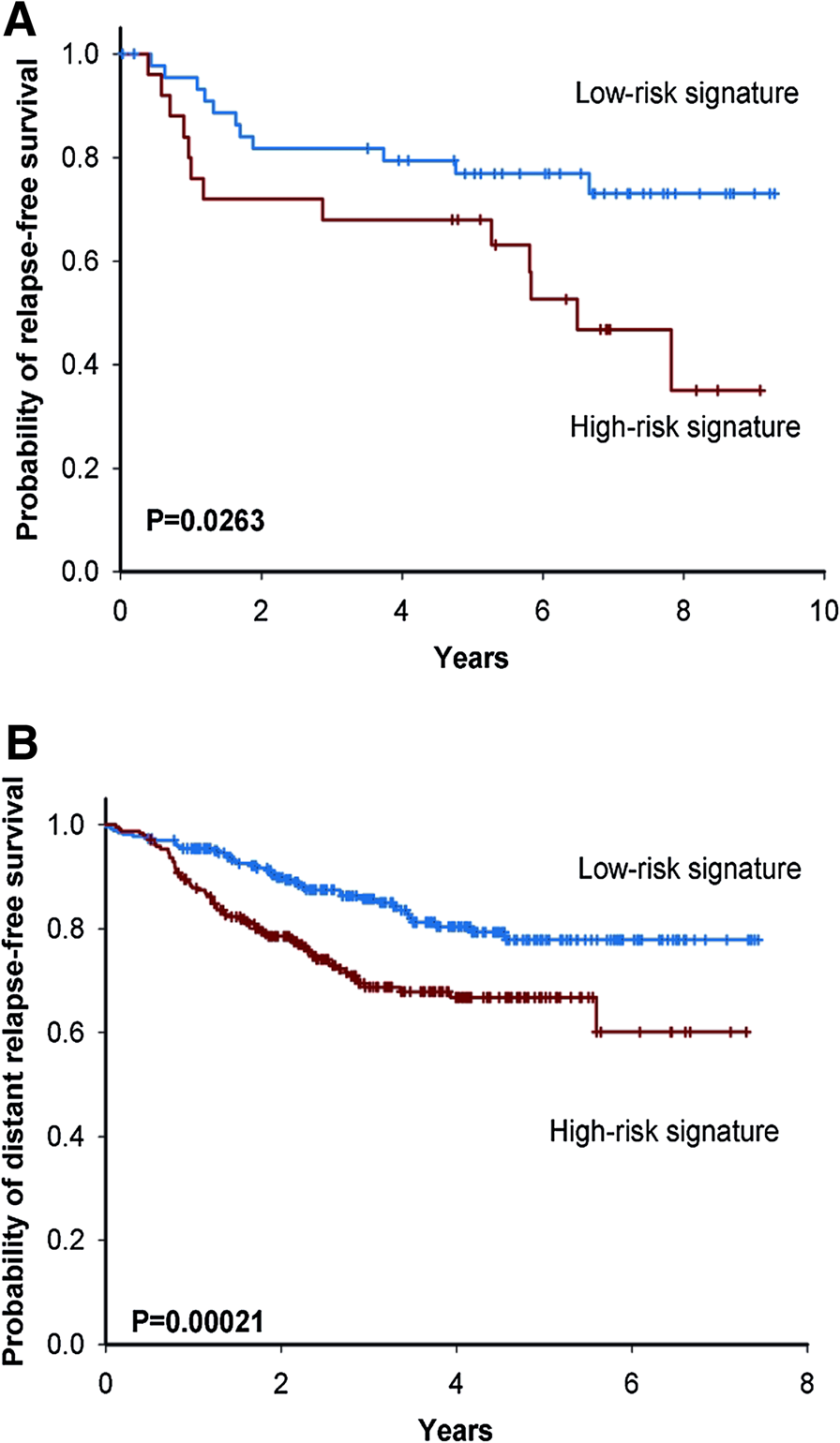
Kaplan–Meier survival curves for survival analysis of the eight-predicted genes in (A) lung cancer and (B) breast cancer cohorts.

We found that the low-risk group has a significantly longer relapse-free survival time than the high-risk group in the lung cancer cohorts (*P* = 0.0263, Figure 7A) and similarly, the low-risk group shows significantly longer distant relapse-free survival in the breast cancer cohorts (*P* = 0.00021, Figure 7B).

We further conducted multivariate Cox proportional hazard regression analysis with our gene signature and other prognostic factors (including age and tumor stage) as the predictors. The result shows that the effect of our gene signature is still significant. The adjusted hazard ratio (HR) is 5.33 (*P* = 0.003) for the lung cancer cohort, and 1.81 (*P* = 0.006) for the breast cancer patients (Table 1). This shows that our eight-gene signature is an independent predictor for patient outcome. To demonstrate that the predicative capacity of the eight-gene signature is specific to chemotherapy, we applied it to four control cohorts (three for breast cancer and one for lung cancer) of which the patients were systemically untreated after surgery. The eight gene signature failed to predict clinical outcome (Additional file 1: Figure S6).

**Table 1:** Multivariate Cox regression analysis of the eight-gene signature for predicting relapse-free survival in cancer patients

**Variable**	**Hazard ratio**	**95% CI**	***P*-value**
**Lung cancer (n = 71)**			
Eight-gene signature	5.33	1.76 to 16.1	**0.003**
Age	1.05	1.00 to 1.11	0.060
Gender (Male vs Female)	1.33	0.48 to 3.68	0. 581
Stage (1 vs 2)	2.69	1.12 to 6.45	**0.027**
Histology Type	1.45	0.56 to 3.73	0.443
**Breast cancer (n = 462)**			
Eight-gene signature	1.81	1.19 to 2.76	**0.006**
Age (>50 vs ≦50)	1.04	0.70 to 1.56	0.834
Clinical nodal status (positive vs negative)	2.47	1.45 to 4.18	**0.0008**
Clinical tumor stage (T3 or T4 vs T1 or T2)	1.80	1.20 to 2.70	**0. 004**
ER status (IHC positive vs negative)	0.44	0.29 to 0.67	**0.0001**

* A total of 46 patients were excluded from the multivariate analysis due to incomplete data in the breast cancer cohort.

## Discussion

The invasive or metastatic potential of a malignant neoplasm and the growth-inhibition of tumor cells by a therapeutic agent are two common denominators of patient survival in cancer systemic therapy. While cell line models have been used to predict treatment response or patient survival by genes associated with drug sensitivity in the cancer cell lines [46-49], these studies have not considered the varying metastasis potential of a tumor. To weigh in the interplay of both factors directly, our pre-clinical gene signature discovery method features the co-integration of invasion phenotypes and compound-sensitity profiles with gene expression at the full genome scale.

Our approach is based on the observation that invasion ability and drug sensitivity are both phenotypes of the cell lines available for study. Each phenotype is naturally associated with its own set of molecular determinants. We hypothesize the potential overlap between the set for invasion ability phenotype and the set for drug responsiveness phenotype. By identifying these common determinants, then we may use these shared determinants to estimate the overall invasion potential of the cancer cells in a tumor and also use it to predict the drug response at the same time. However, because the tumor microenvironment in a patient is different from the growth environment of cancer cell lines monitored in a lab, the robustness of an invasion molecular marker becomes an important factor for increasing the chance of success in clinical applications.

An alternative strategy of analyzing the three-way interaction of invasion, gene expression and drug response would be to correlate invasion with drug response first. Once the most correlated drugs are identified, then genes correlated with response to these drugs can be used to predict the drug sensitivity. However, we did not pursue this line of analysis further because the phenotye-phenotype correlation is often weaker than phenotype-genomic determinant correlation. As a matter of fact, our data show that most drug-invasion correlations appear weak; only four drugs pass the statistical significance and the best two correlations are only 0.39 and −0.35 (Additional file 1: Table S6). Our approach overcomes the limitations of weak phenotype-phenotype correlation by looking for statistical evidence of correlations directly from the genomic determinant. This helps improve the robustness of the genetic marker thus obtained.

Previously, without considering drug sensitivity, our team performed invasion profiling for the nine lung cancer cell lines of NCI-60 to obtain a four gene signature for clinical outcome prediction [50]. We find that among the four genes *ANKRD49* and *LPHN1* are in the IA gene list and only *ANKRD49* has a significant correlation with paclitaxel and docetaxel. The four-gene signature failed to predict the survival outcome for the two validation cohorts receiving adjuvant chemotherapy (Additional file 1: Figure S7).

To gain robustness of our gene signature, instead of using different panels of tissue origins in NCI-60 to obtain different sets of IA genes for different types of cancer, we used the invasion data from all 53 NCI60 solid tumor cell lines and obtained 633 IA genes. Then a series of statistical analyses were designed to increase the robustness of the final eight-gene signature in predicting drug sensitivity for the selected compounds. The eight-gene score differed between drug-resistant and drug-sensitive cell lines (Figure 6). We succeeded in applying our gene signature to one lung cancer cohort and one breast cancer cohort, of which the patients received a regimen containing an anti-microtubule agent. The success in using the same signature to predict patient outcome for different types of cancer showed the robustness of this gene signature.

The eight-gene signature showed a positive correlation with the sensitivity of targeted therapy compounds and a negative correlation with the sensitivity of anti-microtubule compounds (Figure 5B). Because high values of the eight-gene signature correlate with high invasion potential in cancer cells, this suggests that the direct correlation between the invasion profile and the sensitivity profiles of anti-MT drugs may be negative. This is indeed the case, but the correlation is weak (Additional file 1: Table S6, correlation = −0.16, -0.28 for paclitaxel and docetaxel, respectively). On the other hand, the correlation between the eight-gene score and the drug sensitivity is stronger (−0.41, -0.54, respectively). Similarly, the correlation between the eight-gene score and the sensitivity for erlotinib, dasatinib and everolimus is 0.46, 0.52, 0.44, respectively, which is again stronger than the correlation between invasion and drug sensitivity (0.26, 0.24, 0.09, respectively). Therefore, despite the weak correlation between the invasion phenotype and the drug sensitivity phenotype, the eight-gene signature is an effective genomic marker for invasion potential and it can be used to predict the drug’s differential growth-inhibition efficacy that varies between cancer cells of higher invasion potential and those of lower invasion potential.

When applying to the patient’s tumor specimen, the eight-gene signature provides an averaged profile of the gene expression by individual cells with varying invasion potential. A low eight-gene score indicates that the overall invasion potential of the tumor is low and the chance of the patient’s favorable response to regimens containing anti-microtubule compounds increases. On the other hand, a high eight-gene score predicts the abundance of the cells of higher invasion potential, which are harder to eradicate by anti-microtubule compounds, but may be more likely to succumb to the said targeted therapy. This suggests the combined use of targeted therapy like dasatinib or erlotinib with anti-microtubule agents to increase the regimen efficacy of chemotherapy alone. There have been several studies on augmenting the anticancer effect of chemotherapy with targeted therapy. Erlotinib was shown to be more sensitive in the doxorubicin-resistant human breast cancer cell lines and paclitaxel-resistant human ovarian cancer cell lines [51] and the sensitivity was positively correlated with *EGFR* expression. More references were provided in Additional file 1, Supplementary information Text II.

There is room to improve our eight-gene signature for drug-sensitivity prediction. The overlap in distribution between the drug-sensitivity group and the drug-resistant group (Figure 6) suggests that drug response in cell lines is a very complex phenotype which is not fully characterized by our gene signature. Other genomic components, such as DNA copy number, single-nucleotide polymorphisms, methylation and microRNA, have not been considered in our study. In addition, differences in lab environment may also contribute to the variations observed in the data.

## Conclusions

We have shown that augmenting the NCI-60 model with *in vitro* characterization of important phenotypes like invasion potential is a cost-effective approach to power the genomic chemosensitivity analysis. Our analysis delineates the complex three-way interplay of gene expression, cancer cell’s invasion potential and cancer cell’s responsiveness to an anti-cancer compound. We report the identification of a unique eight-gene signature for both lung and breast cancer, which predicted the relapse-free survival of adjuvant chemotherapy patients. The signature features the cancer hallmark *EGFR* and genes involved in cell adhesion cell migration, cell invasion, tumor growth and tumor progression. The discovery of prognostic biomarkers for chemotherapy patients remains critical toward improving the efficacy of cancer treatment. The eight-gene signature obtained here may be useful for the development of individualized cancer therapy. Our method of gene discovery may be applicable in studying other cancers.

## Abbreviations

AHNAK: AHNAK nucleoprotein; Br: Breast cancer; CNS: Central nervous system; CO: Colon cancer; DTP: Development therapeutics program; EGFR: Epidermal growth factor receptor; ER: Estrogen receptor; FAK: Focal adhesion kinase; FDR: False discovery rate; GLS: Glutaminase; HR: Hazard ratio; IA: Invasion-associated; ICC: Invaded cell counts; IL32: Interleukin 32; ITGA3: Integrin, alpha 3; LC: Lung cancer; LIMK1: LIM-kinase 1; ME: Melanoma; MRLC: Myosin regulatory light chains; MYLK: Myosin light chain kinase; NCI: National cancer institute; NNMT: N-methyltransferase; OV: Ovarian cancer; PR: Prostate cancer; RE: Renal cancer.

## Competing interests

The authors declare that they have no competing interests.

## Authors’ contributions

YCH carried out the invasion experiments, conceived the drug sensitivity and clinical prediction study design, and wrote the paper. YCH, HYC, SY, CHL, GW and KCL collected public gene expression and statistical data analysis. SLY participated in setting up the experiment platform for the cell line invasion and qPCR validation. SLY, PCY and KCL provided the study materials and reagents. KCL and YCH set up the study aims and rationalized the approach. PCY and KCL helped chart the study strategy and were involved in drafting and finalizing the manuscript. KCL and YCH proposed the conceptual framework to link gene expression variation with heterogeneity of invasion potential and drug sensitivity in cancer cells. All authors read and approved the final manuscript for publication.

## Pre-publication history

The pre-publication history for this paper can be accessed here:

http://www.biomedcentral.com/1741-7015/11/106/prepub

## Supplementary Material

Additional file 1 **Supplementary information.** Procedures for determining the invasion-associated (IA) genes. II. Combination use of anti-microtubule and targeted therapy agents. **Figure S1.** Histogram of invaded cell counts (ICC) after subtracting the tissue-group means. **Figure S2.** Cell adhesion: Integrin-mediated cell adhesion and migration pathway. **Figure S3.** The heatmap for the correlations between 744 IA gene expression and 99 drug response (−logGI50) in NCI-60 cell lines. **Figure S4.** Validation of microarray gene expression data with qPCR. **Figure S5.** Plots of the eight-gene risk scores between drug sensitive and drug resistant groups of cell lines after removing the nine cell lines that came from the NCI60 panel. The dotted line indicated the mean of each group. **Figure S6.** Kaplan–Meier survival curves for survival analysis of the eight-gene signature in breast and lung cancer patients who did not receive systemic treatment. **Figure S7.** Kaplan–Meier survival curves for survival analysis of the four-gene signature in lung cancer and breast cancer cohorts. **Table S1.** The expression level of eight signature genes in the nine NCI-60 cell lines. **Table S2.** TaqMan probes ID for eight gene expression validation. **Table S3.** Enrichment analysis of 633 invasion-associated genes by functional ontology enrichment tool in MetaCore. **Table S4.** The correlation matrix for (**A**) anti-microtubule and for (**B**) targeted therapy drugs. **Table S5.** Genes having significant gene-drug correlation with everolimus, dasatinib, erlotinib, paclitaxel and docetaxel profiles. The final list of eight IA-genes is shown in bold face. **Table S6.** Correlation between the invasion profile and each of the 99 drug sensitivity profiles of NCI60 cell lines. The significant correlation (*P* <0.05) is shown in bold face. Click here for file
