# Supplementary material for: Genome-wide analysis of three-way interplay among gene expression, cancer cell invasion and anti-cancer compound sensitivity
Source: BMC Med. 2013 Apr 16;11:106. doi: 10.1186/1741-7015-11-106 (PMC3635895; doi:10.1186/1741-7015-11-106)
Supplement: Additional file 1 — Supplementary information. Procedures for determining the invasion-associated (IA) genes. II. Combination use of anti-microtubule and targeted therapy agents. Figure S1. Histogram of invaded cell counts (ICC) after subtracting the tissue-group means. Figure S2. Cell adhesion: Integrin-mediated cell adhesion and migration pathway. Figure S3. The heatmap for the correlations between 744 IA gene expression and 99 drug response (−logGI50) in NCI-60 cell lines. Figure S4. Validation of microarray gene expression data with qPCR. Figure S5. Plots of the eight-gene risk scores between drug sensitive and drug resistant groups of cell lines after removing the nine cell lines that came from the NCI60 panel. The dotted line indicated the mean of each group. Figure S6. Kaplan–Meier survival curves for survival analysis of the eight-gene signature in breast and lung cancer patients who did not receive systemic treatment. Figure S7. Kaplan–Meier survival curves for survival analysis of the four-gene signature in lung cancer and breast cancer cohorts. Table S1. The expression level of eight signature genes in the nine NCI-60 cell lines. Table S2. TaqMan probes ID for eight gene expression validation. Table S3. Enrichment analysis of 633 invasion-associated genes by functional ontology enrichment tool in MetaCore. Table S4. The correlation matrix for (A) anti-microtubule and for (B) targeted therapy drugs. Table S5. Genes having significant gene-drug correlation with everolimus, dasatinib, erlotinib, paclitaxel and docetaxel profiles. The final list of eight IA-genes is shown in bold face. Table S6. Correlation between the invasion profile and each of the 99 drug sensitivity profiles of NCI60 cell lines. The significant correlation (P <0.05) is shown in bold face. [file 1741-7015-11-106-S1.doc]

Supplementary Data

Genome-wide analysis of three-way interplay between gene expression, cancer cell invasion and anti-cancer compound sensitivity

Yi-Chiung Hsu, Hsuan-Yu Chen, Shinsheng Yuan, Sung-Liang Yu, Chia-Hung Lin, Guani Wu, Pan-Chyr Yang, Ker-Chau Li

**Supplementary Information Text**

**I. Procedures for determining the invasion-associated (IA) genes.**

We used HG-U95 to HG-U133 Best Match table available on the Affymetrix website[[1]](#footnote-2) to map U95 probes to the corresponding probes in U133 chip.

**Screening Stage.** Because U133 is the more recent chip design, we used U95 data in the preliminary stage for gene screening. Only the probes with significant Pearson correlation (p value < 0.05) between gene expression and invasion were retained.

**Confirmation stage.** Out of the 4123 probes obtained from U95 after screening, 2315 probes have matched probes in U133. The number of matched probes in U133 was 2417 because a portion of U95 probes had multiple matched probes in U133. We computed the Pearson correlations between the gene expression of these U133 probes and the invasion profile. 831 probes were found to have significant correlations

(p< 0.05). Finally, out of the 831 probes, 744 probes had the same direction of correlation as what the corresponding probes had in the U95 chip. We estimated the false discovery rate in the confirmation stage to be 0.08 (FDR =(2417*0.025)/744).

**II. Combination use of anti-microtubule and targeted therapy agents.**

Several clinical studies indicated that combining targeted therapy with chemotherapy augmented the anti-cancer effects. The combination of dasatinib (Src inhibitor) and paclitaxel had superior activity to either single agent in metastatic breast cancer in the phase I study [1]. The *in vivo* study showed that dasatinib had synergistic activity with paclitaxel, and carboplatin in ovarian cancer cells [2]. Additionally, Src inhibition restored the sensitivity to paclitaxel-resistant ovarian cancer cells and enhanced the anti-cancer effect of paclitaxel by caspase-3 activation [3]. Everolimus (mTOR inhibitor) combined with weekly paclitaxel had encouraging anti-tumor activity in patients with trastuzumab-pretreated and trastuzumab-resistant metastatic HER2-overexpressing breast cancer in the phase I trial [4]. The phase II study indicated that administration of Erlotinib (EGFR TKIs) before carboplatin and paclitaxel improved the response rate and survival in non-small-cell lung cancer (NSCLC) patients [5]. An encouraging response rate in patients with metastatic breast cancer was reported for erlotinib in combination with docetaxel and capecitabine [6]. A recent paper [7] showed an interesting clinical trial outcome. For EGFR wild-type patients, 44 received erlotinib alone and 54 received taxol,carboplatin and erlotinib. The response rate for the former group is 4/44 and the response rate for the latter group is 16/54 (Fisher Exact Test p=0.0131). It has been reported that erlotinib was more sensitive in the doxorubicin-resistant human breast cancer cell line and paclitaxel-resistant human ovarian cancer cell lines [8] and the sensitivity was positively correlated with EGFR expression. Moreover, erlotinib increased the docetaxel accumulation in p-glycoprotein overexpression lung cancer cells [9], all consistent with our results.

**Supplementary Figure S1.** Histogram of invaded cell counts (ICC) after subtracting the tissue-group means. The tissue group means are provided for comparison. “Within-group residual ICC” is the difference between the invaded cell counts for each individual cell-line and its tissue-group average.

**
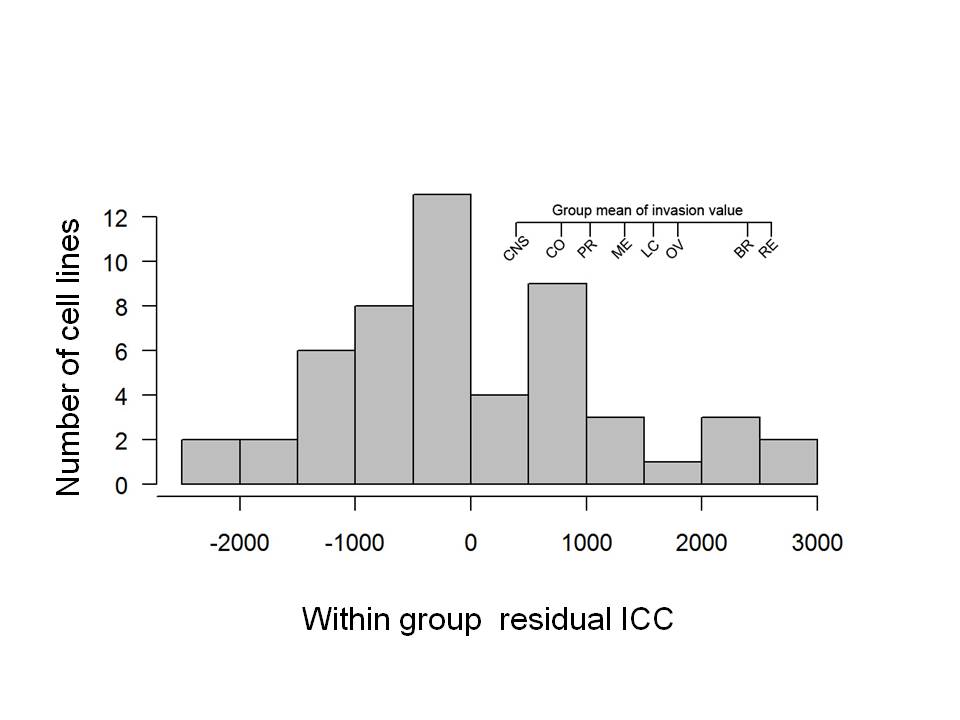
**

**Supplementary Figure S2.** Cell adhesion: Integrin-mediated cell adhesion and migration pathway. Three IA-genes with expression profiles showing positive correlation with the invasion profile and two targeted therapy drugs are marked in red boarders.


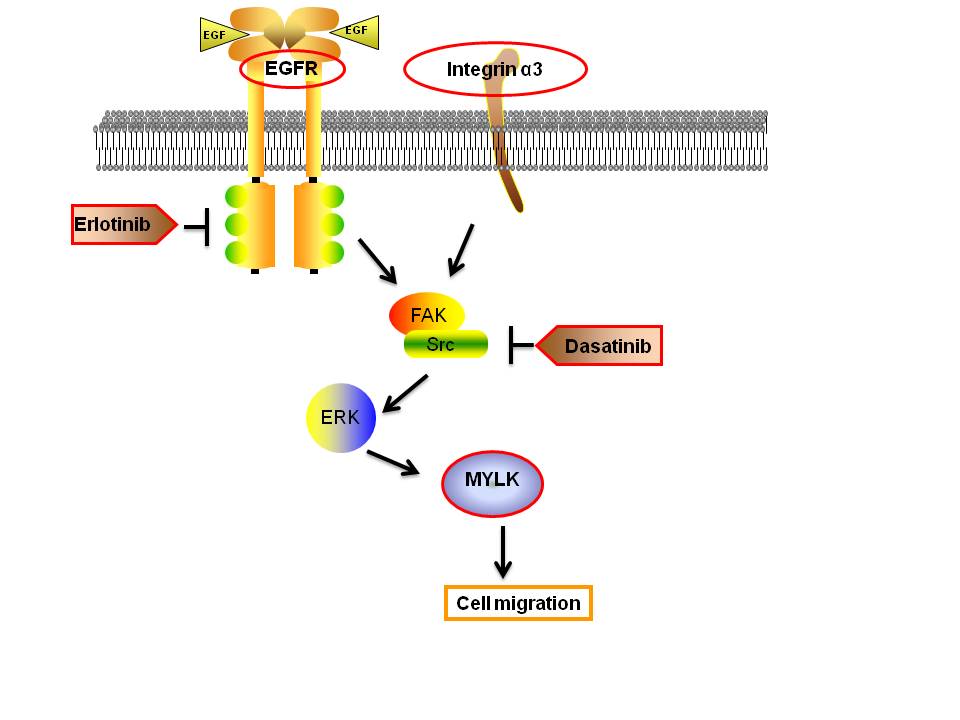


**Supplementary Figure S3.** (A). The heatmap for the correlations between 744 IA gene expression and 99 drug response (-logGI50) in NCI-60 cell lines. Positive correlations were shown in red and negative correlations shown in blue. Color bar the top: Green: tubulin-binding; red: targeted agents; purple: DNA-damaging; orange: hormonal agents; yellow: anti-metabolites; blue: Anthracyclines; black: others.

(B) The drug-sensitivity-correlated IA genes for each of the 99 drugs were shown in blue (negative correlation) or red (positive correlation).

**A.**


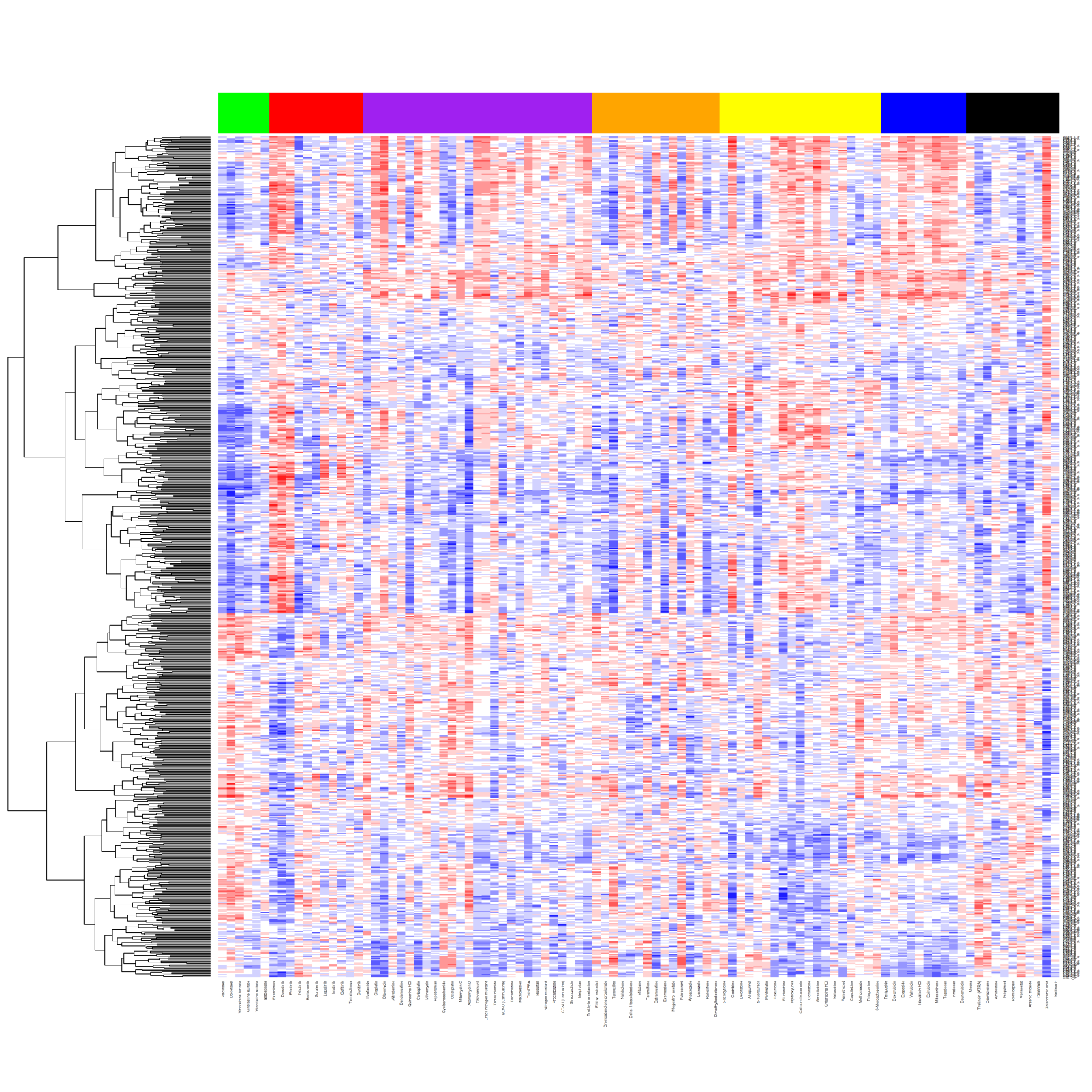


**B.**

**
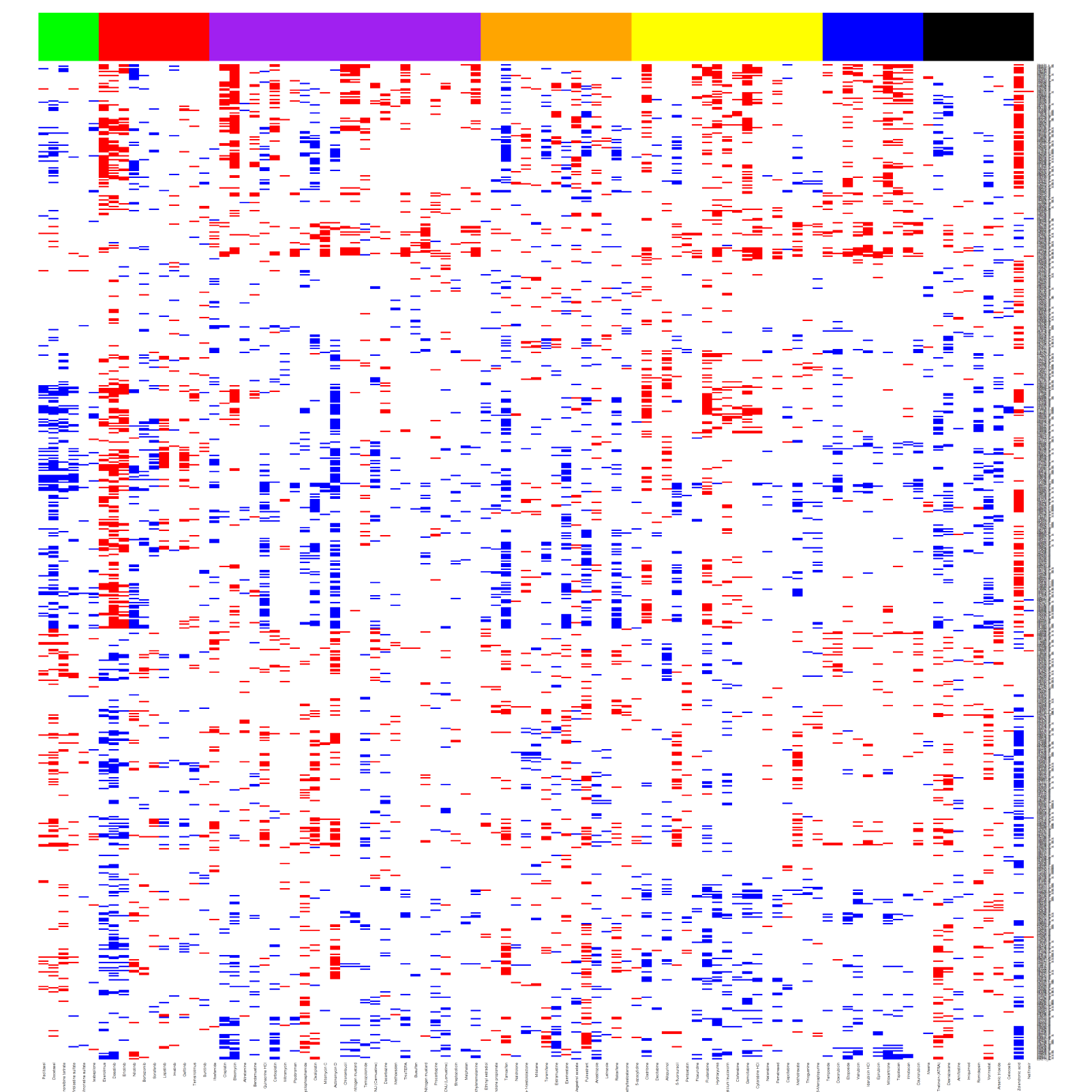
**

**Supplementary Figure S4.** Validation of microarray gene expression data with qPCR.

**Supplementary Figure S5.** Plots of the eight-gene risk scores between drug sensitive and drug resistant groups of cell lines after removing the nine cell lines that came from the NCI60 panel. The dotted line indicated the mean of each group.


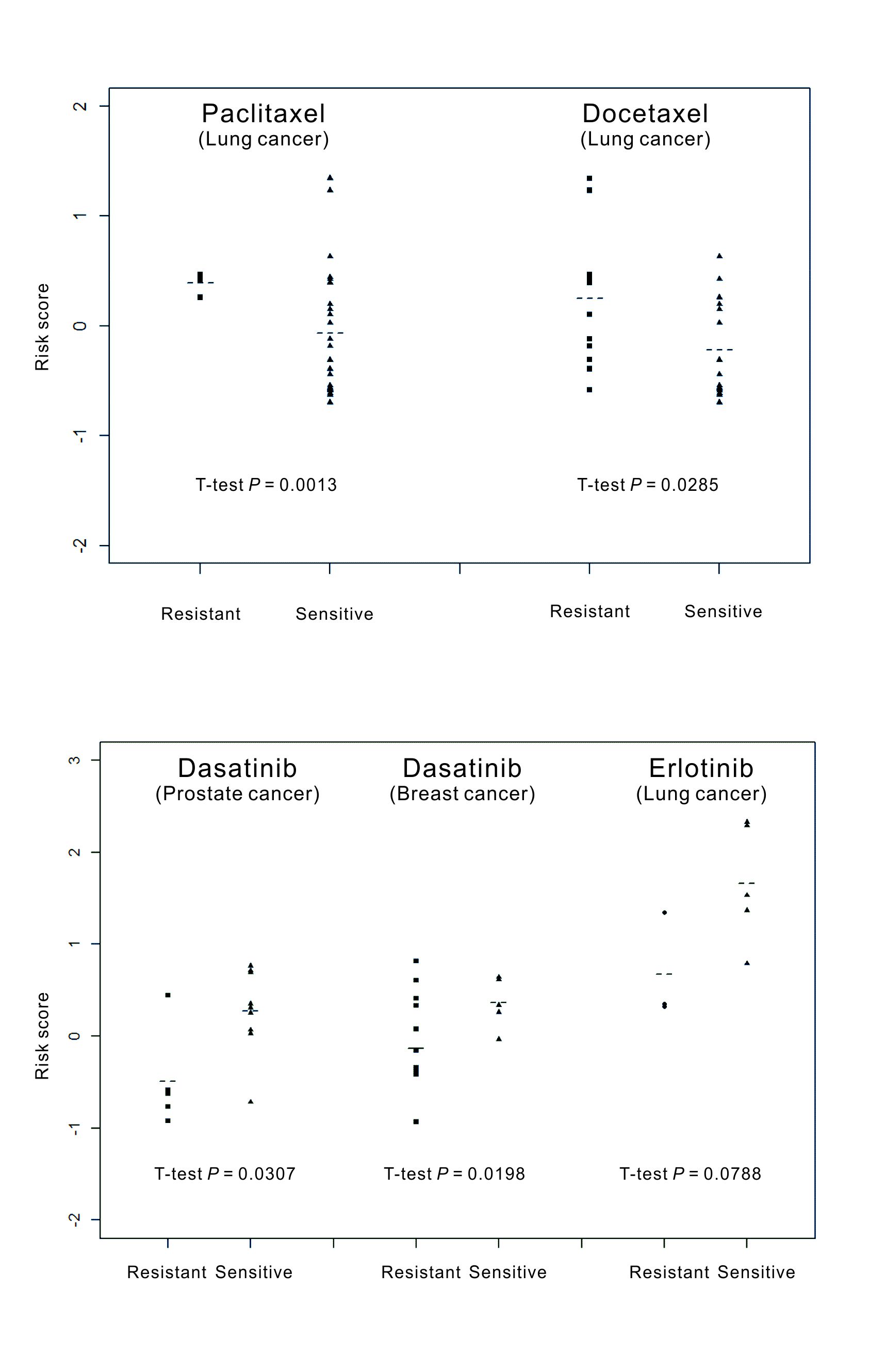


**Supplementary Figure S6.** Kaplan–Meier survival curves for survival analysis of the eight-gene signature in breast and lung cancer patients who did not receive systemic treatment.

**Supplementary Figure S7.** Kaplan–Meier survival curves for survival analysis of the four-gene signature in lung cancer and breast cancer cohorts.

**Supplementary Table S1.** The expression level of eight signature genes in the nine NCI-60 cell lines. These cell lines were contained in the validation group of 78 cell lines. The expression data in Table (A) were generated by different labs and their methods of preprocessing varied. Table (B) showed the data of Affymetrix U133plus2 by Gene Logic. Sensitivity rank (1 being most sensitive) in Table (B) was based on the drug-sensitivity data of the corresponding drug released by NCI.

A.

| Dataset | GSE6569 | GSE6569 | GSE6569 | GSE6569 | Balko et al. | Balko et al. | GSE9633 | GSE9633 | GSE4127 |
| --- | --- | --- | --- | --- | --- | --- | --- | --- | --- |
| Cell line | BR:HS578T | BR:MDA-MB-231 | BR:BT-549 | BR:MCF7 | LC:A549 | LC:NCI-H460 | PR:DU-145 | PR:PC-3 | LC:A549 |
| AHNAK | 11945.3 | 8017.2 | 3032 | 4446.5 | 1512.3 | 680.3 | 8.90907 | 9.47842026 | 2404.5 |
| EGFR | 10697.2 | 13388.2 | 10679.3 | 212.2 | 745.2 | 840.6 | 9.4322004 | 9.08487988 | 3933.4 |
| GLS | 3724.7 | 3530.8 | 2982.5 | 721.4 | 972.1 | 514.1 | 9.8296804 | 8.62979031 | 1005.5 |
| IL32 | 1605.9 | 130.4 | 36.3 | 34.3 | 7.8 | 3.9 | 6.2453098 | 6.88009024 | 217 |
| ITGA3 | 1451.6 | 4812.3 | 1807 | 747.6 | 384.3 | 15.3 | 8.42974 | 8.48705959 | 1848 |
| MYLK | 7328.3 | 2277.4 | 199.9 | 91.6 | 410.9 | 9.2 | 7.6477098 | 6.17643023 | 657.9 |
| NNMT | 8115.3 | 1793.4 | 15415 | 24.1 | 4175.8 | 5.1 | 12.2395 | 8.30873966 | 1677 |
| RAI14 | 2733.7 | 3559.1 | 4876.3 | 1026.9 | 1169.5 | 648.4 | 9.6855602 | 9.89780998 | 1316.5 |
| Normalization | MAS5 | MAS5 | MAS5 | MAS5 | MAS5 | MAS5 | RMA | RMA | MAS5 |
| Drug | Dasatinib | Dasatinib | Dasatinib | Dasatinib | Eroltinib | Eroltinib | Dasatinib | Dasatinib | DOC/PAC |
| Sensitivity | Sensitive | Sensitive | Resistant | Resistant | Resistant | Resistant | Sensitive | Sensitive | Resistant |

B.

| Dataset | NCI-60 | NCI-60 | NCI-60 | NCI-60 | NCI-60 | NCI-60 | NCI-60 | NCI-60 | NCI-60 | NCI-60 |
| --- | --- | --- | --- | --- | --- | --- | --- | --- | --- | --- |
| Gene \Cell line | BR:BT-549 | BR:HS578T | BR:MDA-MB-231 | BR:MCF7 | LC:A549 | LC:NCI-H460 | PR:DU-145 | PR:PC-3 | LC:A549 | LC:A549 |
| AHNAK | 341.67 | 850.91 | 816.25 | 280.29 | 794.54 | 382.18 | 897.82 | 1192.9399 | 794.54 | 794.54 |
| EGFR | 704.75 | 113.87 | 120.91 | 3.35 | 328.38 | 153.68 | 605.73 | 372.46 | 328.38 | 328.38 |
| GLS | 61.26 | 344.89 | 199.35 | 33.28 | 94.88 | 98.78 | 486.31 | 137.63 | 94.88 | 94.88 |
| IL32 | 2.46 | 330.52 | 97.85 | 4.47 | 22.62 | 6.61 | 35.82 | 6.93 | 22.62 | 22.62 |
| ITGA3 | 151.19 | 58.47 | 477.1 | 43.75 | 146.44 | 20.11 | 260.15 | 198.34 | 146.44 | 146.44 |
| MYLK | 38.38 | 259.64 | 106.69 | 5.1 | 138.48 | 3.9 | 90.82 | 10.08 | 138.48 | 138.48 |
| NNMT | 1132.37 | 433.81 | 118.53 | 4.2 | 221.1 | 77.5 | 714.32 | 12.96 | 221.1 | 221.1 |
| RAI14 | 348.98 | 218.81 | 493.92 | 62.94 | 157.25 | 80.79 | 284.62 | 268.48 | 157.25 | 157.25 |
| Drug | Dasatinib | Dasatinib | Dasatinib | Dasatinib | Erlotinib | Erlotinib | Dasatinib | Dasatinib | Paclitaxel | Docetaxel |
| Sensitivity rank | 49/53 | 1/53 | 10/53 | 50/53 | 30/53 | 21/53 | 26/53 | 29/53 | 17/53 | 18/53 |

**Supplementary Table S2.** TaqMan probes ID for eight gene expression validation

| Gene Name |  | Assay ID |  |
| --- | --- | --- | --- |
| AHNAK | Hs01102461_m1 | | |
| TBP | Hs99999910_m1 | | |
| NNMT | Hs00196287_m1 | | |
| EGFR | Hs01076078_m1 | | |
| MYLK | Hs00364926_m1 | | |
| IL32 | Hs00170403_m1 | | |
| GLS | Hs00248163_m1 | | |
| RAI14 | Hs00210238_m1 | | |
| ITGA3 | Hs01076873_m1 | | |

| **Supplementary Table S3.** Enrichment analysis of 633 invasion-associated genes by functional ontology enrichment tool in MetaCore | | | |
| --- | --- | --- | --- |
| **GeneGo Pathway Maps** | | | |
| Pathway name | P-Value | Input nodes | Total nodes |
| Cytoskeleton remodeling_Cytoskeleton remodeling | 3.35589E-11 | 19 | 102 |
| Cell adhesion_Chemokines and adhesion | 2.04393E-10 | 18 | 100 |
| Cell adhesion_Integrin-mediated cell adhesion and migration | 5.48886E-08 | 11 | 48 |
| Cytoskeleton remodeling_TGF, WNT and cytoskeletal remodeling | 5.87501E-08 | 16 | 111 |
| Cytoskeleton remodeling_Regulation of actin cytoskeleton by Rho GTPases | 1.07698E-07 | 8 | 23 |
| Cell adhesion_Role of tetraspanins in the integrin-mediated cell adhesion | 5.33485E-07 | 9 | 37 |
| Development_Regulation of epithelial-to-mesenchymal transition (EMT) | 1.22044E-06 | 11 | 64 |
| Development_TGF-beta-dependent induction of EMT via RhoA, PI3K and ILK. | 3.79878E-06 | 9 | 46 |
| Cytoskeleton remodeling_Role of PKA in cytoskeleton reorganization | 1.12258E-05 | 8 | 40 |
| Development_Slit-Robo signaling | 1.37717E-05 | 7 | 30 |
| **GeneGo Process Networks** | | | |
| Network name | P-Value | Input nodes | Total nodes |
| Cytoskeleton_Actin filaments | 1.44896E-15 | 35 | 176 |
| Cytoskeleton_Regulation of cytoskeleton rearrangement | 5.12239E-15 | 35 | 183 |
| Cell adhesion_Integrin-mediated cell-matrix adhesion | 8.31488E-11 | 32 | 214 |
| Cell adhesion_Cell junctions | 4.88193E-07 | 22 | 162 |
| Development_EMT_Regulation of epithelial-to-mesenchymal transition | 6.32384E-06 | 25 | 232 |
| Cell adhesion_Cadherins | 1.05599E-05 | 21 | 180 |
| Cytoskeleton_Intermediate filaments | 1.86555E-05 | 13 | 81 |
| Immune response_Phagocytosis | 3.06372E-05 | 23 | 223 |
| Cell adhesion_Platelet aggregation | 7.04836E-05 | 19 | 174 |

**Supplementary Table S4.** The correlation matrix for (A) anti-micortubule and for (B) targeted therapy drugs. The correlation between two columns of the heatmap Figure 5A is shown in this table. The selected drugs were shown in bold face.

A.

| Targeted agents | **Everolimus** | **Dasatinib** | **Erlotinib** | Nilotinib | Bortezomib | Sorafenib | Lapatinib | Imatinib | Gefitinib | Temsirolimus | Sunitinib |
| --- | --- | --- | --- | --- | --- | --- | --- | --- | --- | --- | --- |
| **Everolimus** | **1** | **0.79550848** | **0.792960822** | -0.68191073 | -0.4443618 | -0.65280926 | 0.300113722 | -0.44944085 | 0.156956124 | 0.686733077 | -0.22550181 |
| **Dasatinib** | **0.7955** | **1** | **0.797647781** | -0.63336789 | -0.44796098 | -0.55729627 | 0.221445304 | -0.382651 | 0.140517437 | 0.622632816 | -0.11840595 |
| **Erlotinib** | **0.793** | **0.797647781** | **1** | -0.6561275 | -0.52332292 | -0.71237 | 0.394332865 | -0.63549493 | 0.347007662 | 0.501224783 | -0.20081181 |
| Nilotinib | -0.6819 | -0.633367891 | -0.6561275 | 1 | 0.544760944 | 0.304728612 | 0.060034035 | 0.503097194 | 0.20269143 | -0.49166568 | 0.361502669 |
| Bortezomib | -0.4444 | -0.447960977 | -0.52332292 | 0.544760944 | 1 | 0.381208528 | -0.14032509 | 0.496050404 | 0.008622392 | -0.2310516 | 0.188371118 |
| Sorafenib | -0.6528 | -0.55729627 | -0.71237 | 0.304728612 | 0.381208528 | 1 | -0.63925847 | 0.578388321 | -0.47773629 | -0.42251253 | 0.080294219 |
| Lapatinib | 0.3001 | 0.221445304 | 0.394332865 | 0.060034035 | -0.14032509 | -0.63925847 | 1 | -0.31852102 | 0.828684566 | 0.102956983 | 0.220043803 |
| Imatinib | -0.4494 | -0.382650999 | -0.63549493 | 0.503097194 | 0.496050404 | 0.578388321 | -0.31852102 | 1 | -0.22140683 | -0.22892406 | 0.361241602 |
| Gefitinib | 0.157 | 0.140517437 | 0.347007662 | 0.20269143 | 0.008622392 | -0.47773629 | 0.828684566 | -0.22140683 | 1 | 0.010651661 | 0.197940053 |
| Temsirolimus | 0.6867 | 0.622632816 | 0.501224783 | -0.49166568 | -0.2310516 | -0.42251253 | 0.102956983 | -0.22892406 | 0.010651661 | 1 | -0.19208587 |
| Sunitinib | -0.2255 | -0.118405954 | -0.20081181 | 0.361502669 | 0.188371118 | 0.080294219 | 0.220043803 | 0.361241602 | 0.197940053 | -0.19208587 | 1 |

B.

| Tubulin-biding agents | **Paclitaxel** | **Docetaxel** | Vinorelbine | Vinblastine | Vincristine | Ixabepilone |
| --- | --- | --- | --- | --- | --- | --- |
| **Paclitaxel** | **1** | **0.87458175** | 0.786609648 | 0.841893936 | 0.771733854 | 0.659865408 |
| **Docetaxel** | **0.8746** | **1** | 0.656917703 | 0.704262844 | 0.638806672 | 0.524528138 |
| Vinorelbine | 0.7866 | 0.656917703 | 1 | 0.749536341 | 0.496726936 | 0.40138939 |
| Vinblastine | 0.8419 | 0.704262844 | 0.749536341 | 1 | 0.660999234 | 0.636114529 |
| Vincristine | 0.7717 | 0.638806672 | 0.496726936 | 0.660999234 | 1 | 0.60232222 |
| Ixabepilone | 0.6599 | 0.524528138 | 0.40138939 | 0.636114529 | 0.60232222 | 1 |

| **Supplementary Table S5.** Genes having significant gene-drug correlation with everolimus, dasatinib, erlotinib, paclitaxel and docetaxel profiles. The final list of eight IA-genes is shown in bold face. | | | | | | | | | | | | | | | | | |
| --- | --- | --- | --- | --- | --- | --- | --- | --- | --- | --- | --- | --- | --- | --- | --- | --- | --- |
| **Probe ID** | **GENE_SYM** | **Gene name** | | | | **Docetaxel** | | **Paclitaxel** | | **Dasatinib** | | **Erlotinib** | | **Everolimus** | | **Platform** | **SD Rank** |
| **211986_at** | **AHNAK** | **AHNAK nucleoprotein** | | | | -0.39602 | | -0.36132 | | 0.526879 | | 0.492707 | | 0.374114 | | U133A | 952 |
| **202237_at** | **NNMT** | **nicotinamide N-methyltransferase** | | | | -0.38598 | | -0.34482 | | 0.326784 | | 0.302557 | | 0.335597 | | U133A | 1200 |
| **201983_s_at** | **EGFR** | **epidermal growth factor receptor** | | | | -0.37672 | | -0.31948 | | 0.371385 | | 0.408162 | | 0.406985 | | U133A | 1429 |
| **202555_s_at** | **MYLK** | **myosin, light chain kinase** | | | | -0.45975 | | -0.42827 | | 0.430946 | | 0.273881 | | 0.450214 | | U133A | 2469 |
| **203828_s_at** | **IL32** | **interleukin 32** | | | | -0.34358 | | -0.30253 | | 0.416306 | | 0.322338 | | 0.323504 | | U133A | 3030 |
| **221510_s_at** | **GLS** | **glutaminase** | | | | -0.32226 | | -0.36694 | | 0.358547 | | 0.378366 | | 0.29911 | | U133A | 3260 |
| **202052_s_at** | **RAI14** | **retinoic acid induced 14** | | | | -0.46813 | | -0.39003 | | 0.288024 | | 0.273434 | | 0.306513 | | U133A | 3488 |
| **201474_s_at** | **ITGA3** | **integrin, alpha 3** | | | | -0.52239 | | -0.41312 | | 0.445851 | | 0.365446 | | 0.319718 | | U133A | 3684 |
| 204798_at | MYB | v-myb myeloblastosis viral oncogene homolog (avian) | | | | 0.424571 | | 0.392162 | | -0.41005 | | -0.49802 | | -0.32529 | | U133A | 1393 |
| 218606_at | ZDHHC7 | zinc finger, DHHC-type containing 7 | | | | -0.37944 | | -0.31073 | | 0.475457 | | 0.332998 | | 0.647439 | | U133A | 5106 |
| 202822_at | LPP | LIM domain containing preferred translocation partner in lipoma | | | | -0.44488 | | -0.31119 | | 0.453269 | | 0.356978 | | 0.29369 | | U133A | 5733 |
| 218627_at | DRAM | damage-regulated autophagy modulator | | | | -0.37837 | | -0.44238 | | 0.320253 | | 0.502282 | | 0.435046 | | U133A | 6022 |
| 213429_at | NULL | NULL | | | | -0.39332 | | -0.38985 | | 0.410057 | | 0.405004 | | 0.456477 | | U133A | 7489 |
| 202552_s_at | CRIM1 | cysteine rich transmembrane BMP regulator 1 (chordin-like) | | | | -0.3291 | | -0.35944 | | 0.356056 | | 0.307835 | | 0.293923 | | U133A | 8193 |
| 210094_s_at | PARD3 | par-3 partitioning defective 3 homolog (C. elegans) | | | | -0.37983 | | -0.30153 | | 0.372488 | | 0.422412 | | 0.416042 | | U133A | 8521 |
|  | | |  |  |  | |  | |  | |  | |  | |  | | |
| **Probe ID** | **GENE_SYM** | **Gene name** | | | | **Docetaxel** | | **Paclitaxel** | | **Dasatinib** | | **Erlotinib** | | **Everolimus** | | **Platform** | **SD Rank** |
| 201984_s_at | EGFR | epidermal growth factor receptor (erythroblastic leukemia viral (v-erb-b) oncogene homolog, avian) | | | | -0.38921 | | -0.3183 | | 0.392715 | | 0.37363 | | 0.273316 | | U133A | 8660 |
| 217910_x_at | MLX | MAX-like protein X | | | | 0.320337 | | 0.330011 | | -0.2826 | | -0.40414 | | -0.42521 | | U133A | 16115 |
| 210879_s_at | RAB11FIP5 | RAB11 family interacting protein 5 (class I) | | | | -0.37922 | | -0.33713 | | 0.364362 | | 0.370163 | | 0.329192 | | U133A | 16812 |
| 207034_s_at | GLI2 | GLI-Kruppel family member GLI2 | | | | -0.41598 | | -0.37467 | | 0.298258 | | 0.282088 | | 0.395425 | | U133A | 29262 |
| 224302_s_at | MRPS36 | mitochondrial ribosomal protein S36 | | | | 0.502529 | | 0.27413 | | -0.3944 | | -0.30932 | | -0.45233 | | U133B | 2571 |
| 224870_at | KIAA0114 | KIAA0114 | | | | 0.313628 | | 0.321922 | | -0.40725 | | -0.29056 | | -0.3445 | | U133B | 621 |
| 224999_at | NULL | NULL | | | | -0.42747 | | -0.30393 | | 0.366105 | | 0.383604 | | 0.33631 | | U133B | 1171 |
| 225406_at | TWSG1 | twisted gastrulation homolog 1 (Drosophila) | | | | -0.32569 | | -0.3119 | | 0.344269 | | 0.362584 | | 0.277105 | | U133B | 3135 |
| 225688_s_at | PHLDB2 | pleckstrin homology-like domain, family B, member 2 | | | | -0.4084 | | -0.30503 | | 0.284459 | | 0.302121 | | 0.311393 | | U133B | 755 |
| 226282_at | NULL | Full length insert cDNA clone ZE03F06 | | | | -0.31106 | | -0.30297 | | 0.464021 | | 0.355004 | | 0.392821 | | U133B | 927 |
| 228537_at | GLI2 | GLI-Kruppel family member GLI2 | | | | -0.38845 | | -0.29168 | | 0.338795 | | 0.282315 | | 0.331244 | | U133B | 8742 |

**Supplementary Table S6.** Correlation between the invasion profile and each of the 99 drug sensitivity profiles of NCI60 cell lines. The significant correlation (p<0.05) is shown in bold face.

| Mechanism | Drug name | correlation | P.val |
| --- | --- | --- | --- |
| Target therapy | Bortezomib | -0.131756034 | 0.346994728 |
| Target therapy | Temsirolimus | 0.016444569 | 0.906961965 |
| Target therapy | Gefitinib | -0.05070427 | 0.718426399 |
| Target therapy | Erlotinib | 0.094426048 | 0.501236714 |
| Target therapy | Dasatinib | 0.240949492 | 0.085300315 |
| Target therapy | Everolimus | 0.262448739 | 0.057627124 |
| Target therapy | Imatinib | 0.003097417 | 0.98243848 |
| Target therapy | Lapatinib | 0.00389472 | 0.978138634 |
| Target therapy | Nilotinib | 0.043912017 | 0.754879416 |
| Target therapy | Sorafenib | -0.024169285 | 0.864950987 |
| Target therapy | Sunitinib | 0.134122883 | 0.338324286 |
| Tubulin-directed agents | Paclitaxel | -0.156646454 | 0.262653139 |
| Tubulin-directed agents | Vinblastine sulfate | -0.071442393 | 0.611203189 |
| Tubulin-directed agents | Vinorelbine tartrate | -0.081018421 | 0.571955539 |
| Tubulin-directed agents | Docetaxel | -0.279853175 | 0.072657046 |
| Tubulin-directed agents | Vincristine sulfate | -0.02139705 | 0.879127175 |
| Tubulin-directed agents | Ixabepilone | -0.05797612 | 0.686133174 |
| Anthracyclines/Topoisomerase poisons | Teniposide | -0.038778699 | 0.782789985 |
| Anthracyclines/Topoisomerase poisons | Doxorubicin | -0.14163797 | 0.311698828 |
| Mechanism | Drug name | correlation | P.val |
| Anthracyclines/Topoisomerase poisons | Etoposide | 0.122603674 | 0.381796616 |
| Anthracyclines/Topoisomerase poisons | Valrubicin | 0.021924465 | 0.878628022 |
| Anthracyclines/Topoisomerase poisons | Idarubicin HCl | -0.158391758 | 0.266944461 |
| Anthracyclines/Topoisomerase poisons | Epirubicin | 0.055529288 | 0.701708015 |
| Anthracyclines/Topoisomerase poisons | Mitoxantrone | 0.02145588 | 0.878797327 |
| Anthracyclines/Topoisomerase poisons | Topotecan | 0.053282829 | 0.704743905 |
| Anthracyclines/Topoisomerase poisons | Irinotecan | -0.009556318 | 0.945854397 |
| Anthracyclines/Topoisomerase poisons | Daunorubicin | -0.104669182 | 0.455736659 |
| Antimetabolites/Nucleosides | 5-azacytidine | 0.078572585 | 0.575995933 |
| Antimetabolites/Nucleosides | Cladribine | 0.292279553 | 0.060344182 |
| Antimetabolites/Nucleosides | Decitabine | 0.17086637 | 0.221222422 |
| Antimetabolites/Nucleosides | Allopurinol | 0.063281565 | 0.652592927 |
| Antimetabolites/Nucleosides | 5-fluorouracil | -0.037837572 | 0.787937735 |
| Antimetabolites/Nucleosides | Pentostatin | -0.150177435 | 0.283118817 |
| Mechanism | Drug name | correlation | P.val |
| Antimetabolites/Nucleosides | Floxuridine | 0.064928119 | 0.644152681 |
| **Antimetabolites/Nucleosides** | **Fludarabine** | **0.279572655** | **0.042620356** |
| Antimetabolites/Nucleosides | Hydroxyurea | 0.119267948 | 0.394979683 |
| Antimetabolites/Nucleosides | Calcium leucovorin | 0.242505157 | 0.12177069 |
| Antimetabolites/Nucleosides | Clofarabine | 0.14145293 | 0.322114282 |
| Antimetabolites/Nucleosides | Gemcitabine | 0.223448513 | 0.107758801 |
| Mechanism | Drug name | correlation | P.val |
| Antimetabolites/Nucleosides | Cytarabine HCl | 0.201362378 | 0.14822172 |
| Antimetabolites/Nucleosides | Nelarabine | 0.013915814 | 0.922000844 |
| Antimetabolites/Nucleosides | Pemetrexed | 0.190272253 | 0.176663658 |
| Antimetabolites/Nucleosides | Capcitebine | -0.155687002 | 0.280307482 |
| Antimetabolites/Nucleosides | Methotrexate | -0.086567703 | 0.537661328 |
| Antimetabolites/Nucleosides | Thioguanine | 0.026683583 | 0.849574819 |
| Antimetabolites/Nucleosides | 6-Mercaptopurine | -0.041985141 | 0.765321863 |
| **DNA damaging agents** | **Ifosfamide** | **-0.355300221** | **0.009034759** |
| DNA damaging agents | Cisplatin | -0.015060261 | 0.914763621 |
| DNA damaging agents | Bleomycin | 0.086083049 | 0.539949241 |
| DNA damaging agents | Altretamine | -0.195745314 | 0.16011124 |
| DNA damaging agents | Bendamustine | 0.066970737 | 0.633743852 |
| DNA damaging agents | Quinacrine HCl | -0.172595371 | 0.216513982 |
| DNA damaging agents | Carboplatin | -0.014658734 | 0.917028065 |
| DNA damaging agents | Mitramycin | -0.241534436 | 0.081444129 |
| DNA damaging agents | Pipobroman | -0.005355603 | 0.969639913 |
| DNA damaging agents | Cyclophosphamide | -0.227836502 | 0.100842567 |
| DNA damaging agents | Oxaliplatin | -0.119104071 | 0.395634143 |
| DNA damaging agents | Mitomycin C | -0.082867307 | 0.555248485 |
| DNA damaging agents | Actinomycin D | -0.181345472 | 0.19375881 |
| DNA damaging agents | Chlorambucil | -0.004893235 | 0.972259932 |
| Mechanism | Drug name | correlation | P.val |
| DNA damaging agents | Uracil nitrogen mustard | 0.044198248 | 0.753331877 |
| DNA damaging agents | Temozolomide | 0.207084962 | 0.14074281 |
| DNA damaging agents | BCNU (Carmustine) | -0.155370907 | 0.266607903 |
| DNA damaging agents | Dacarbazine | 0.040268054 | 0.774662451 |
| DNA damaging agents | Methoxsalen | -0.022054375 | 0.892550698 |
| DNA damaging agents | ThioTEPA | 0.066305652 | 0.637125427 |
| DNA damaging agents | Busulfan | -0.116213086 | 0.40728378 |
| DNA damaging agents | Nitrogen mustard | -0.127494909 | 0.362946662 |
| **DNA damaging agents** | **Procarbazine** | **0.283625829** | **0.039583977** |
| DNA damaging agents | CCNU (Lomustine) | 0.180502387 | 0.195873997 |
| DNA damaging agents | Streptozoticin | 0.020755737 | 0.882724157 |
| DNA damaging agents | Melphalan | -0.011170345 | 0.936726686 |
| DNA damaging agents | Triethylenemelamine | 0.031363905 | 0.823580012 |
| Hormonal agents | Ethinyl estradiol | -0.142925592 | 0.372683894 |
| Hormonal agents | Dromostanolone propionate | -0.017612506 | 0.900386463 |
| Hormonal agents | Tamoxifen | -0.15664083 | 0.26267049 |
| Hormonal agents | Naldrolone | -0.012756009 | 0.927767213 |
| Hormonal agents | Delta-1-testololactone | 0.103081993 | 0.462635732 |
| Hormonal agents | Mitotane | 0.226150288 | 0.103458559 |
| Hormonal agents | Toremifene | -0.129493669 | 0.38037192 |
| Hormonal agents | Estramustine | 0.096288006 | 0.497109794 |
| Mechanism | Drug name | correlation | P.val |
| Hormonal agents | Exemestane | -0.154724337 | 0.273419291 |
| Hormonal agents | Megestrol acetate | 0.072165105 | 0.611170396 |
| Hormonal agents | Fulvestrant | -0.109983233 | 0.433051829 |
| Hormonal agents | Anastrozole | 0.238973445 | 0.105718769 |
| Hormonal agents | Letrozole | 0.073213206 | 0.613361819 |
| Hormonal agents | Raloxifene | -0.041906738 | 0.765747646 |
| Hormonal agents | Dimethyltestosterone | -0.142246569 | 0.30960296 |
| Other | Mesna | -0.133377363 | 0.355798292 |
| Other | Tretinoin (ATRA) | -0.066419577 | 0.636545659 |
| Other | Dexrazoxane | -0.117478258 | 0.402161363 |
| Other | Amifostine | 0.279850493 | 0.072659902 |
| Other | Imiquimod | 0.082969951 | 0.55475699 |
| Other | Romidepsin | -0.054074614 | 0.700560786 |
| Other | Vorinostat | -0.183609993 | 0.188158095 |
| Other | Arsenic trioxide | -0.239113836 | 0.08463961 |
| Other | Celecoxib | -0.193548946 | 0.178058587 |
| **Other** | **Zolendronic acid** | **0.39385073** | **0.003524597** |
| Other | Nelfinavir | -0.026756149 | 0.849170496 |

References:

1. Fornier MN, Morris PG, Abbruzzi A, D'Andrea G, Gilewski T, Bromberg J, Dang C, Dickler M, Modi S, Seidman AD, Sklarin N, Chang J, Norton L, Hudis CA: **A phase I study of dasatinib and weekly paclitaxel for metastatic breast cancer.** *Ann Oncol* 2011.

2. Teoh D, Ayeni TA, Rubatt JM, Adams DJ, Grace L, Starr MD, Barry WT, Berchuck A, Murphy SK, Secord AA: **Dasatinib (BMS-35482) has synergistic activity with paclitaxel and carboplatin in ovarian cancer cells.** *Gynecol Oncol* 2011, **121:**187-192.

3. Chen T, Pengetnze Y, Taylor CC: **Src inhibition enhances paclitaxel cytotoxicity in ovarian cancer cells by caspase-9-independent activation of caspase-3.** *Mol Cancer Ther* 2005, **4:**217-224.

4. Andre F, Campone M, O'Regan R, Manlius C, Massacesi C, Sahmoud T, Mukhopadhyay P, Soria JC, Naughton M, Hurvitz SA: **Phase I study of everolimus plus weekly paclitaxel and trastuzumab in patients with metastatic breast cancer pretreated with trastuzumab.** *J Clin Oncol* 2010, **28:**5110-5115.

5. Riely GJ, Rizvi NA, Kris MG, Milton DT, Solit DB, Rosen N, Senturk E, Azzoli CG, Brahmer JR, Sirotnak FM, Seshan VE, Fogle M, Ginsberg M, Miller VA, Rudin CM: **Randomized phase II study of pulse erlotinib before or after carboplatin and paclitaxel in current or former smokers with advanced non-small-cell lung cancer.** *J Clin Oncol* 2009, **27:**264-270.

6. Twelves C, Trigo JM, Jones R, De Rosa F, Rakhit A, Fettner S, Wright T, Baselga J: **Erlotinib in combination with capecitabine and docetaxel in patients with metastatic breast cancer: a dose-escalation study.** *Eur J Cancer* 2008, **44:**419-426.

7. Janne PA, Wang X, Socinski MA, Crawford J, Stinchcombe TE, Gu L, Capelletti M, Edelman MJ, Villalona-Calero MA, Kratzke R, Vokes EE, Miller VA: **Randomized phase II trial of erlotinib alone or with carboplatin and paclitaxel in patients who were never or light former smokers with advanced lung adenocarcinoma: CALGB 30406 trial.** *J Clin Oncol* 2012, **30:**2063-2069.

8. Dai Q, Ling YH, Lia M, Zou YY, Kroog G, Iwata KK, Perez-Soler R: **Enhanced sensitivity to the HER1/epidermal growth factor receptor tyrosine kinase inhibitor erlotinib hydrochloride in chemotherapy-resistant tumor cell lines.** *Clin Cancer Res* 2005, **11:**1572-1578.

9. Collins DM, Crown J, O'Donovan N, Devery A, O'Sullivan F, O'Driscoll L, Clynes M, O'Connor R: **Tyrosine kinase inhibitors potentiate the cytotoxicity of MDR-substrate anticancer agents independent of growth factor receptor status in lung cancer cell lines.** *Invest New Drugs* 2010, **28:**433-444.

1. <http://www.affymetrix.com/support/technical/byproduct.affx?product=hgu133> [↑](#footnote-ref-2)
